# Overlapping regions of Caf20 mediate its interactions with the mRNA-5′cap-binding protein eIF4E and with ribosomes

**DOI:** 10.1038/s41598-021-92931-4

**Published:** 2021-06-29

**Authors:** Ebelechukwu C. Nwokoye, Eiman AlNaseem, Robert A. Crawford, Lydia M. Castelli, Martin D. Jennings, Christopher J. Kershaw, Graham D. Pavitt

**Affiliations:** 1 Division of Molecular and Cellular Function, School of Biological Sciences, Faculty of Biology, Medicine and Health, Manchester Academic Health Science Centre, The University of Manchester, Manchester, M13 9PT UK; 2 Department of Botany, Nnamdi Azikiwe University, Awka, Nigeria; 3 Present Address: Department of Neuroscience, Sheffield Institute for Translational Neuroscience (SITraN), University of Sheffield, Sheffield, S10 2HQ UK

**Keywords:** Biochemistry, Molecular biology, Translation, Ribosome

## Abstract

By interacting with the mRNA 5′ cap, the translation initiation factor eIF4E plays a critical role in selecting mRNAs for protein synthesis in eukaryotic cells. Caf20 is a member of the family of proteins found across eukaryotes termed 4E-BPs, which compete with eIF4G for interaction with eIF4E. Caf20 independently interacts with ribosomes. Thus, Caf20 modulates the mRNA selection process via poorly understood mechanisms. Here we performed unbiased mutagenesis across Caf20 to characterise which regions of Caf20 are important for interaction with eIF4E and with ribosomes. Caf20 binding to eIF4E is entirely dependent on a canonical motif shared with other 4E-BPs. However, binding to ribosomes is weakened by mutations throughout the protein, suggesting an extended binding interface that partially overlaps with the eIF4E-interaction region. By using chemical crosslinking, we identify a potential ribosome interaction region on the ribosome surface that spans both small and large subunits and is close to a known interaction site of eIF3. The function of ribosome binding by Caf20 remains unclear.

## Introduction

In eukaryotic cells mRNAs are typically modified by the addition of a 5′ 7-methyl-guanosine cap and a 3′ poly(A) tail. Efficient initiation of protein synthesis is dependent on these features^1^. A 43S pre-initiation complex (PIC) containing initiator tRNA, a 40S ribosomal subunit and specific eukaryotic initiation factors (eIFs) is directed to bind near the mRNA 5′ cap, before it scans to locate the AUG start codon, via mRNA codon-initiator tRNA anti-codon base pairing^2–4^. The 5′ cap is bound by the translation factor eIF4E, to which eIF4G binds. This complex promotes recruitment of the PIC to capped mRNAs and represses translation from uncapped mRNAs^5^. That this eIF4G-eIF4E interaction is critical for promoting PIC-mRNA interactions, is also indicated by the action of eIF4G competitor proteins collectively called eIF4E-binding proteins (4E-BPs), that are found in all eukaryotes studied from yeasts to man. eIF4G and the 4E-BPs share an overlapping interaction site on the surface of eIF4E and all possess a common short ‘canonical’ sequence (YX_4_LΦX_2_K/R, where Φ is a hydrophobic residue) which makes direct contact with eIF4E^6–9^.

The binding affinity of several 4E-BPs to eIF4E is modulated through phosphorylation of the 4E-BP outside the canonical binding motif^6^. For example, phosphorylation of serines and threonines both before and after the cannonical motif within mammalian proteins 4E-BP1 and 2 combine to prevent 4E-BP–eIF4E interaction in response to growth-promoting signalling through mTOR^10^. A reduction in 4E-BP–eIF4E binding facilitates the eIF4G–eIF4E interaction and promotes protein synthesis. Phosphorylation of 4E-BP2 was shown to dramatically promote its folding into a domain that effectively prevents the canonical motif binding to eIF4E^11^. Other structural studies examining the interactions of extended peptides from several 4E-BPs with eIF4E have found that binding of the canonical motif region to eIF4E is supplemented by additional surrounding sequences that also make contact with the eIF4E surface. Specifically the region after the canonical helix can form an ‘elbow loop’ followed by a non-canonical (NC) helix which contribute to the binding affinity to eIF4E as observed in mammalian 4E-BP1 and 4E-T, *Drosophila* Thor and CUP, as well as yeast Eap1^7–9^.

The yeast *Saccharomyces cerevisiae* possesses two 4E-BPs Caf20 (also known as p20)^12^ and Eap1^13^ that both possess canonical eIF4E binding sequences, but which share no other clear homology with metazoan 4E-BPs. Although similar in size to 4E-BP1/2 and *Drosophila* Thor, Caf20 appears atypical because its canonical eIF4E motif is at its extreme amino terminus (residues 4–13). Unlike 4E-BP1/2 control, no clear growth-rate regulation of Caf20 phosphorylation or eIF4E interaction change has been observed, making it unclear how eIF4E–Caf20 binding is controlled. By performing a series of affinity purification of TAP-tagged protein factors and RNA sequencing we identified mRNAs enriched with the 5′ cap complex proteins, Caf20 and Eap1and the polyA-binding protein Pab1. The patterns of mRNA enrichments identified four broad groups of mRNAs each enriched with a different set of factors^14^. Consistent with Caf20 and Eap1 having roles as translational repressors, enriched mRNAs were found to be relatively long and have a lower density of translating ribosomes. The broad number of enriched mRNAs was consistent with Caf20 and Eap1 having a wide set of mRNA targets. Curiously, one group of mRNAs was enriched with Caf20 and/or Eap1 but not by eIF4E, suggesting each 4E-BP may bind mRNAs independently of eIF4E interaction^14^. Further analysis identified that 3′ UTRs of several mRNAs were able to bind Caf20, directly or indirectly, independent of its eIF4E-binding activity^15^. A subsequent separate study showed that a purified Caf20–eIF4E complex bound with higher affinity than eIF4E alone to 5′ capped RNA and that the Caf20 complex also bound uncapped RNA^16^. We also found that Caf20 can co-fractionate with ribosomes and again this interaction was independent of its ability to bind to eIF4E^15^. Curiously, Altmann and colleagues found that Caf20 could enhance translation in in vitro translation extracts programmed with specific mRNAs^16^. These observations suggest that Caf20 may function with binding partners in addition to eIF4E and have a more complex role in translation.

In this study we have undertaken a molecular biology approach to evaluate features of Caf20 required for its interaction with eIF4E and with ribosomes. We made a series of Caf20 plasmids each deleted for a specific region and evaluated the protein’s ability to interact with its binding partners. We find the amino terminal canonical motif is the only element critical for eIF4E interaction, while ribosome binding relies on an extended region of Caf20. Then we use a chemical cross-linking approach to further identify where on ribosomes Caf20 may interact. We find and evaluate three potential ribosomal protein partners found on one side of the ribosome away from the main tRNA and translation factor binding sites.

## Results

### Caf20 binds tightly to eIF4E through its amino terminus

**Figure 1:**
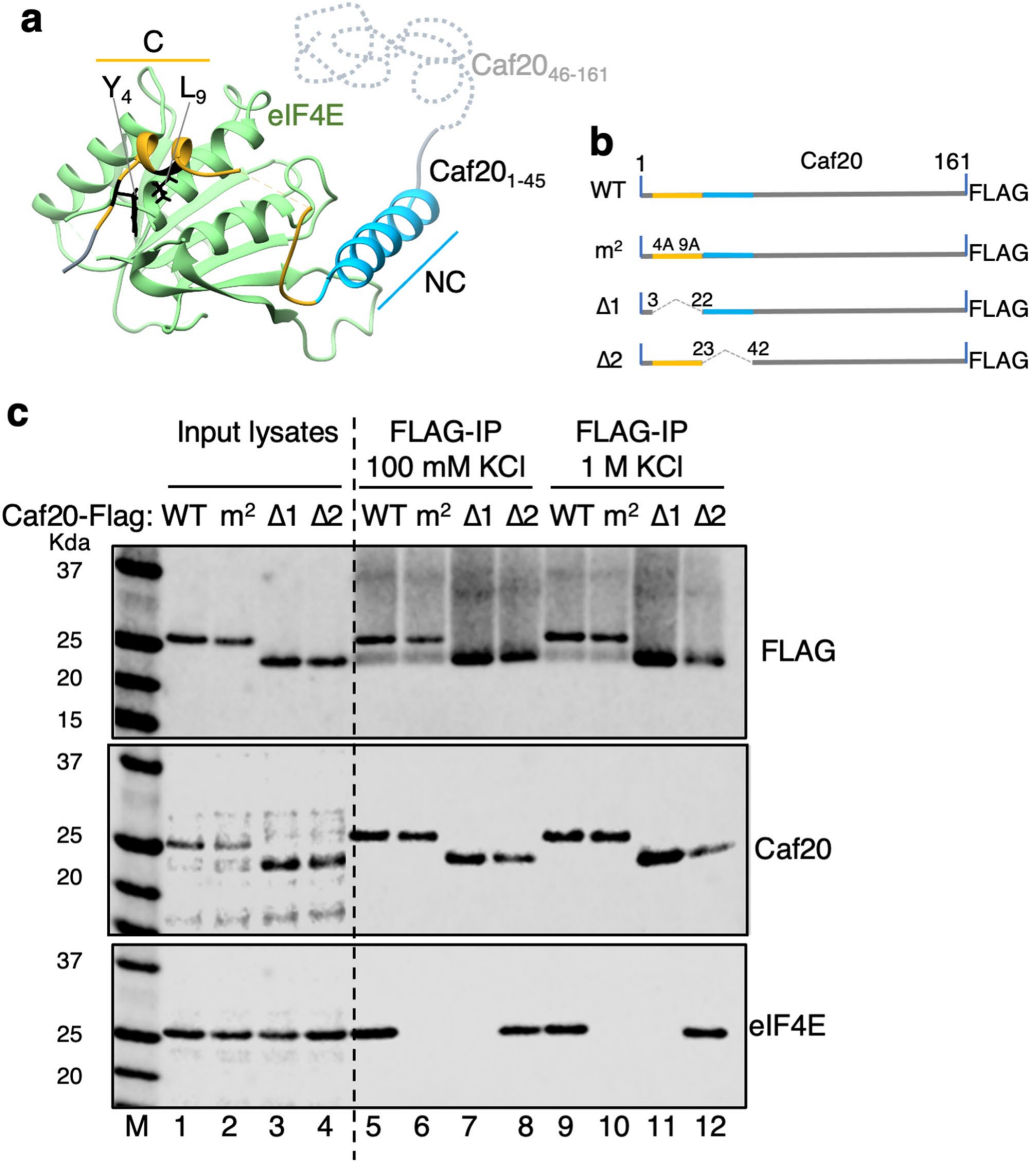
Caf20 binds tightly to eIF4E, except when canonical interaction is eliminated. (**a**) Caf20^1–45^ interacts with eIF4E via 2 alpha helices. Cartoon from PDB 6FC3 (ref 9) with canonical (C) and non-canonical helices interacting with eIF4E. (**b**) Cartoon of Flag tagged Caf20 showing m^2^ and deletion alleles each removing 20 amino acids. (**c**) Western blots showing expression in yeast extracts (lanes 1–4) and immunoprecipitation of Caf20-FLAG (Top and middle) and native eIF4E (bottom) with Flag resin (lanes 5–12). Lanes 5–8 IPs washed with 100 mM KCl, lane 9–12 1 M. Note: faint bands in lanes 5, 6, 9, 10 that co-migrate with Caf20^∆1^ represent cross-reactivity with M2 Flag antibody light-chain co-eluted from resin (so are absent when probed with an antibody to the C-terminus of Caf20, middle panel).

Recently the structure of the amino terminal third (residues 1–45) of Caf20 bound to yeast eIF4E was determined^9^. This showed that in addition to the conserved shared canonical 4E-BP motif (YX_4_LΦX_2_K/R) found at the very amino terminus of Caf20 a second so-called non-canonical helix (NC) also makes extensive contact with the eIF4E surface (Fig. 1a). Biochemical studies of the interaction between this Caf20 peptide and eIF4E allowed the authors to conclude that tight binding-between Caf20 and eIF4E was mediated largely by the canonical helix (*K*_D_ = 20 nM, by calorimetry). Other 4E-BPs tested, including Eap1 were found to rely more on surrounding elements including the NC helix to stabilize the interaction, despite their overall similar binding strategies^9^. Earlier work, where full-length Caf20 interaction with eIF4E was studied, concluded that eIF4G bound with tenfold enhanced affinity over Caf20^17^. We therefore decided to re-evaluate Caf20-eIF4E interactions in the context of the full-length protein expressed in yeast cells. We expressed Caf20-FLAG from a plasmid in *caf20∆* cells and evaluated its binding to eIF4E using Flag-affinity resin (Fig. 1b,c). As expected from prior studies, eIF4E bound to Caf20, but not to Caf20-FLAG bearing a double missense mutation Y4A, L9A that removes two conserved residues critical to the canonical binding helix and defined as the Caf20^m2^ mutation^15,18^. We extended this analysis in two ways, first by independently deleting either the canonical or the NC helices to create the Caf20^∆1^ and Caf20^∆2^ mutants, each removing 20 amino acids from the full-length protein (Fig. 1b) and evaluating their binding to eIF4E. Secondly by using a high salt buffer (1 M KCl) in the binding assay, we evaluated if there were changes caused by an altered ionic strength (Fig. 1c and Fig. S1). These experiments showed that in the context of proteins expressed in native yeast cells, Caf20-eIF4E interactions are stable to these stringent binding conditions, but entirely dependent on the canonical binding helix at the N-terminus of Caf20.

**Figure 2:**
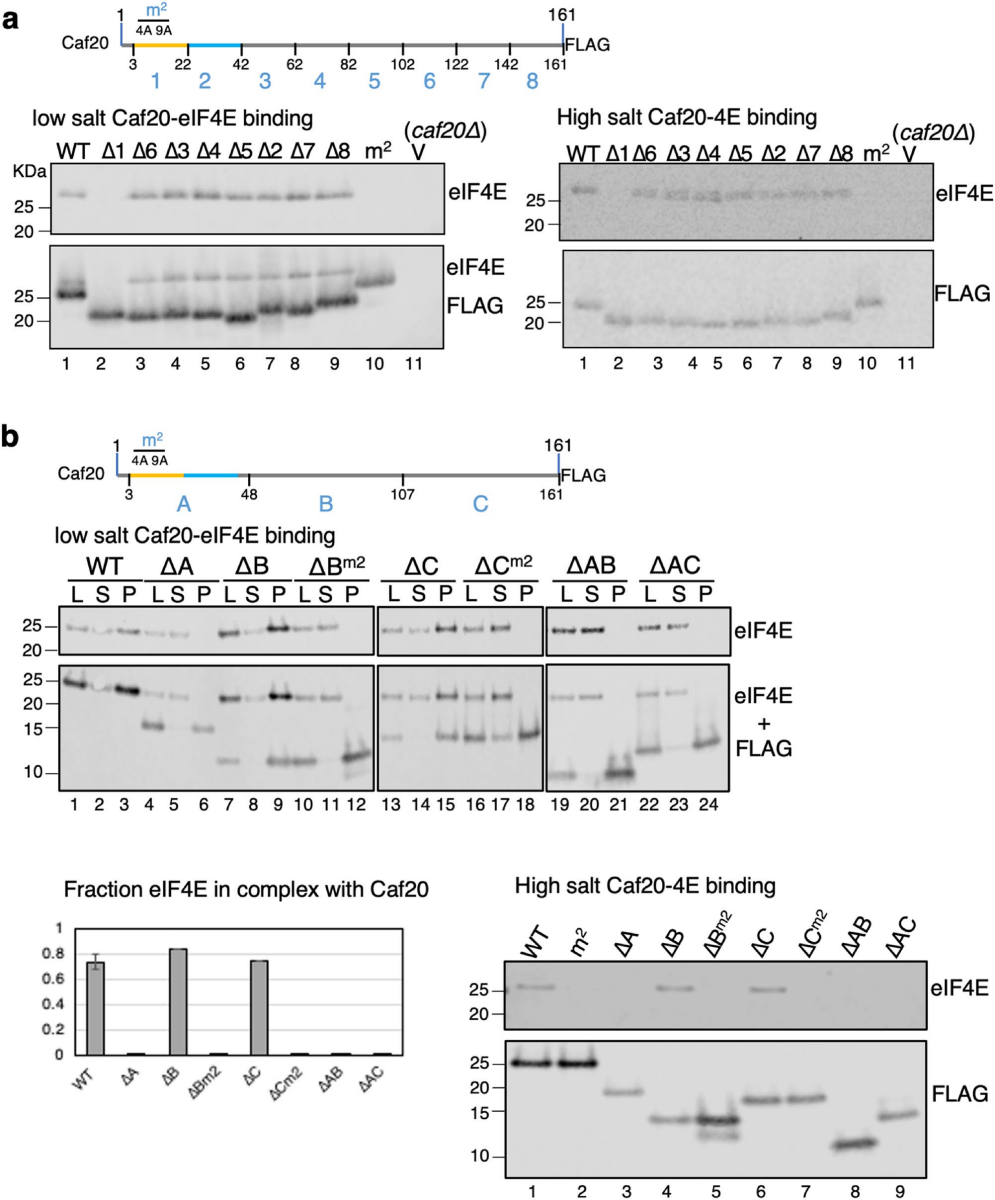
The canonical-binding motif is the only critical element in Caf20 for Binding to eIF4E. Co-immunoprecipitation of eIF4E with *CAF20*-FLAG deleted for specific regions with eIF4E from cell protein extracts. (**a**) 20-residue deletions ∆1–∆8. Western blots of Flag peptide eluted eIF4E and Flag-tagged Caf20 from total cell extracts. On the left, IP washed in low salt buffer (100 mM KCl). On the right, western blots of Caf20-FLAG co-IPs performed as in the left panel except that pellets were washed in high salt buffer (1 M KCl). (**b**) Co-immunoprecipitation as in panel (**a**), but with larger elements removed. Top cartoon summarises the deletions. Western panels show Input (load, L), Unbound supernatant (S) and 3xFlag peptide eluted Pellets (P) for samples washed with 100 mM KCl. Lower panels show quantification of eIF4E bound (left) and 3xFlag peptide eluted fraction only for equivalent experiments with 1 M KCl buffer (right). Experiments performed 2–3 times with equivalent results.

To assess if any other regions of Caf20 modulated binding to eIF4E we made some further deletion mutants. Firstly we extended our series of 20-residue deletions through Caf20 creating mutants ∆3-∆8. Secondly we made a series of larger deletions using an alignment of related sequences and secondary structure predictions (Fig. S2). We divided Caf20 into three regions A-C, where region A comprised the N-terminus (1–48) as used for structural studies and regions B and C represented the middle and C-terminus. We made nine constructs each deleting one or two of these three regions. Where region A was retained we additionally made deletion constructs with the m^2^ mutations. Western blotting with anti-Flag anitisera confirmed all additional mutant forms were well expressed in yeast (Fig. S3) with the exception of mutant ∆BC and its m^2^ version which only retain region A, equivalent to that used for earlier structural studies (Fig. S3b, lanes 9 and 10). These two mutants were not used again. A series of Flag immunopreciptitation experiments was performed using soluble protein extracts from cells expressing each of these mutants as the sole source of Caf20 at both low and high salt (Fig. 2). These experiments clearly showed that all mutants bound well to eIF4E, except those where the canonical helix at the extreme amino terminus was either missing (∆1 or ∆A) or contained the m^2^ amino acid substitutions. No clear reduction in or enhanced binding to eIF4E was observed for any other mutant combination. Thus, perhaps surprisingly the Caf20-eIF4E interaction is entirely dependent on the Caf20 amino terminal helix and the remaining parts of Caf20 do not appear to impact eIF4E interactions.

### An extended region of Caf20 promotes its interaction with ribosomes

It was demonstrated previously by a variety of assays that Caf20 binds directly or indirectly to ribosomes independently from its interaction with eIF4E^15^. By mass spectrometry (MS), ribosomal proteins were found associated with Caf20 and by polysome gradient fractionation analysis Caf20 was distributed across polyribosome-associated and ribosome free fractions^15^. The Caf20^m2^ mutant was similarly associated with ribosomes, implying that Caf20-ribosome interactions were independent of its ability to bind to eIF4E. Ribosome association was also resistant to RNase I treatment, implying that any Caf20-mRNA interaction was likely not important for 80S ribosome binding. In contrast the 4E-BP Eap1 was not polysome associated, and was found mainly in lighter fractions with some in the 80S peak^15^.

**Figure 3:**
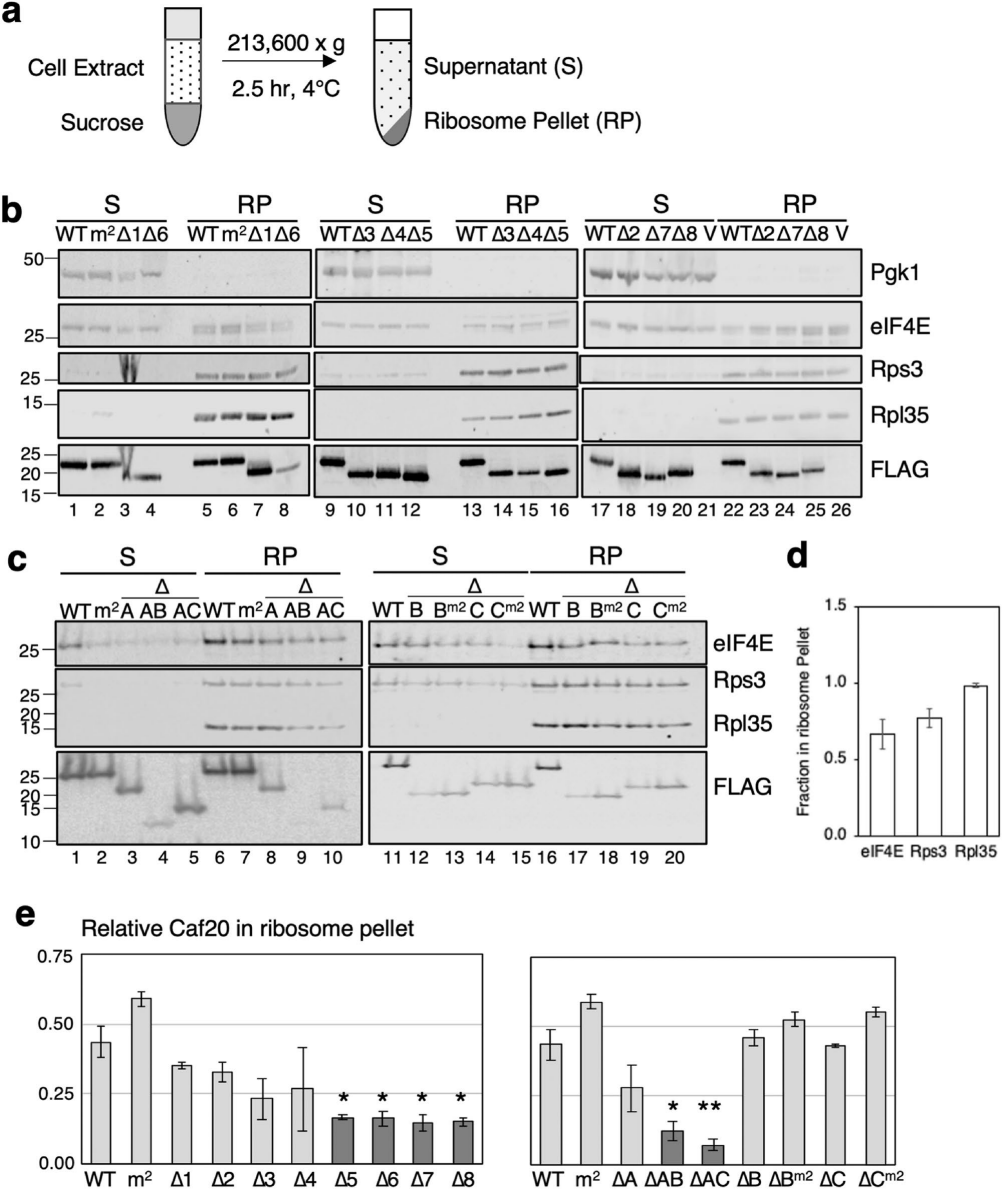
Caf20 mutants reduce ribosome binding. (**a**) Sucrose cushion and ultracentrifugation technique used to separate extract into supernatant and pellet fractions. (**b**) and (**c**) Western blots of supernatant (S) and ribosome pellet (RP) fractions of soluble proteins from cell lysates from strains bearing indicated Caf20 alleles. (**d**) and (**e**) Quantification of fraction of western blot signals in RP fractions (± SE). T-test (1-tailed) for significance *p < 0.05, **p < 0.01 (n = 2–9) for samples compared to WT.

To identify if any part of Caf20 was necessary for ribosome association we performed a sucrose cushion fractionation to separate cell extracts into ribosome bound and free fractions (Fig. 3a). Although this approach results in a lower resolution analysis than full-polysome fractionation, it enabled the full set of Caf20 deletion mutants to be evaluated side-by-side. We used antibodies to Rps3 and Rpl35 to track ribosomes and Pgk1 antibodies to mark supernatant fractions (S) (Fig. 3b–d). Across experiments approximately 67% of eIF4E was found in the ribosome pellet fraction, which was not dependent on Caf20 (Fig. 3d). In agreement with previous studies both WT and m2 mutant forms of Caf20 associated equally with ribosomes. In contrast to eIF4E associations, there were differences in association found with Caf20 mutants. Specifically, each small deletion ∆5-∆8 which removed 20 residues from the c-terminal half of Caf20 reduced RP association by twofold (Fig. 3b,e). In contrast larger deletion ∆C which removes residues deleted in the smaller ∆6–8 constructs had no statistically significant impact on binding (Fig. 3c) unless region A was also removed (∆AC). It remains unclear why the smaller deletions (∆5–8) have a larger impact than ∆C, perhaps the new sequence junctions created interfere with an unknown structural element that has greater impact than when the C-terminus is eliminated. Similar observations were made comparing ∆A, ∆B and ∆AB. The data suggest that unlike binding to eIF4E, there is no single discreet binding site on Caf20 that mediates ribosome interaction, instead multiple regions along Caf20 contribute to ribosome binding. When only the central region, residues 48–108, was retained (∆AC) ribosome binding was the most deficient.

Both rates of colony formation and global polyribosome profiles of cells bearing Caf20 variants where eIF4E interaction was eliminated and/or ribosome binding impaired did not reveal any clear global effects (Fig. S4). These results are consistent with the idea that removing a repressor of specific mRNAs does not impact growth or global translation by this measure^15^.

### Caf20 can be cross-linked to multiple proteins including eIF4E

**Figure 4:**
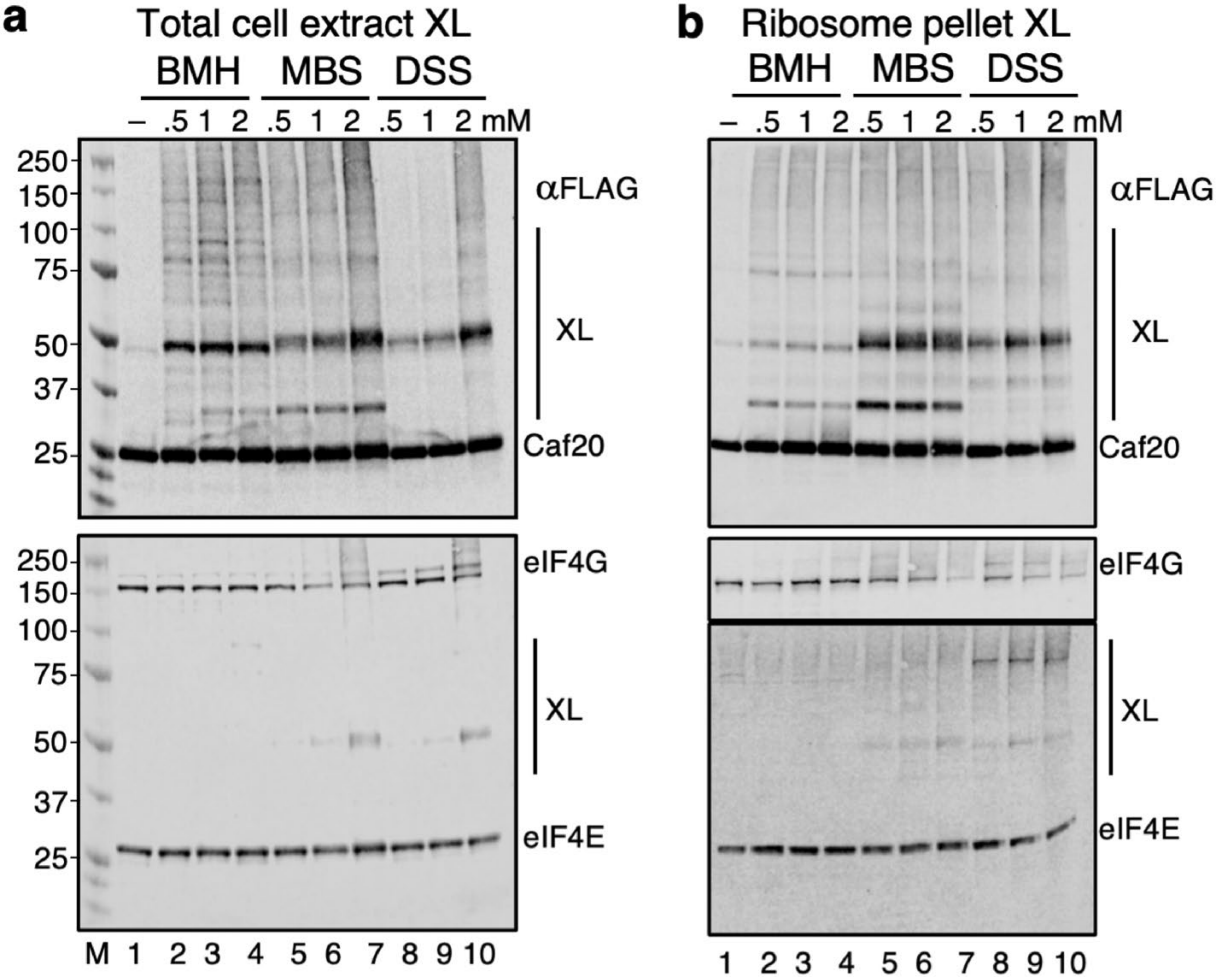
Caf20 crosslinks to proteins associated with ribosomes. (**a**) Western blotting whole cell extracts from Caf20-Flag cells following treatment with BMH, MBS and DSS (0.5, 1 or 2 mM). Probed with Flag, eIF4G and eIF4E antibodies. (**b**) As panel a except for samples enriched in ribosomes by sucrose cushion. lanes 1 ‘–’ samples treated with DMSO solvent only.

As Caf20 binds to the ribosome independently of eIF4E (Fig. 3b), we decided to explore whether we could use chemical cross-linking (XL) to identify Caf20 interacting partners in cell extracts. Three different crosslinkers were evaluated: bismaleimidohexane (BMH), which covalently links between two cysteine residue sulfhydral groups, m-maleimidobenzoyl-N-hydroxysuccinimide ester (MBS) links amine-to sulfhydryl groups (lysine to cystine) and disuccinimidyl suberate (DSS) links primary amine groups (eg lysine to lysine). Cell extracts from Caf20-FLAG cells were mixed with increasing concentrations of each crosslinker and reaction products were resolved by SDS-PAGE and western blotting (Fig. 4a). Each crosslinker appeared to react with Caf20 as evidenced by the appearance of more slowly migrating XL-specific bands when imaged with anti-Flag antibodies. Probing with anti-eIF4E revealed similar retarded species for both BMS and DSS, but not BMH, indicating that the eIF4E XL band at approximately 50 KDa was likely an eIF4E-Caf20 species which agrees with the sum of their masses. Hence Caf20 appears to XL to additional proteins giving rise to major bands at approximately 35 and 50 KDa. Caf20 has a single cysteine and eIF4E has no cysteines which is consistent with BMH failing to link eIF4E. The single cys in Caf20 is at residue 82 (underlined in Fig. S2a), outside our Caf20 region A which was used for the earlier structure determination^9^. The BMH spacer arm is 13 Å, which places the middle of Caf20 in relatively close proximity with the eIF4E amino terminus or a surface lysine amine group of which there are multiple candidates.

### Caf20 interacting partners are enriched with ribosomes

To identify whether any Caf20 XL interacting partners were ribosome associated, we combined our ribosome-pelleting approach (Fig. 3a) with XL to conjugate proteins closest to the fraction of Caf20-FLAG associated with ribosomes. The results obtained revealed that the major Caf20 XL partners were also enriched in ribosome pellets (Fig. 4b).

As one of the major XL partners was predicted to be the same size as Caf20-FLAG itself, we investigated if Caf20 could form a stable dimer. We expressed Caf20-FLAG plasmids in a strain where the endogenous *CAF20* was tagged with 9xMyc and performed αFlag IP. No Caf20-myc was recovered in the pellets (Fig. S5), suggesting stable Caf20 dimers do not form. The same conclusion was drawn from further control XL experiments. We used MBS to XL cells extracts from untagged, Flag, Myc and TAP-tagged. Each tag shifted Caf20 by different amounts, but the major XL bands were 10 and 25 KDa heavier in each case (Fig. S6a, lanes 1–4). These data show that the XL species are not Caf20 dimers and not related to the tag.

XL products could also be detected in pellet fractions following tag immunoprecipitation experiments (Fig. S6a, lanes 8–10). XL products were also independent of eIF4E interaction because Caf20 and Caf20^m2^ XL were equivalent when using MBS (Fig. S6b) or BMH (Fig. S6d). As expected, cysteine 82 was necessary for crosslinking with the sulfhydryl-dependent XL MBS or BMH, as Caf20^∆4^ which removed this residue did not generate any Caf20 XL (Fig. S6c,d), but retains eIF4E and ribosome interactions. In contrast, extracts from a strain expressing only Caf20^∆AC^, which significantly reduced ribosome binding but retains cysteine 82, exhibited an altered XL pattern that did not enrich proteins with masses of approximately 10 and 25 KDa. In summary these experiments demonstrate that Caf20 cysteine 82 can be crosslinked to eIF4E and to other unidentified proteins that are enriched in ribosome pellets.

### Mass spectrometry identifies Caf20 interaction partner targets

**Figure 5:**
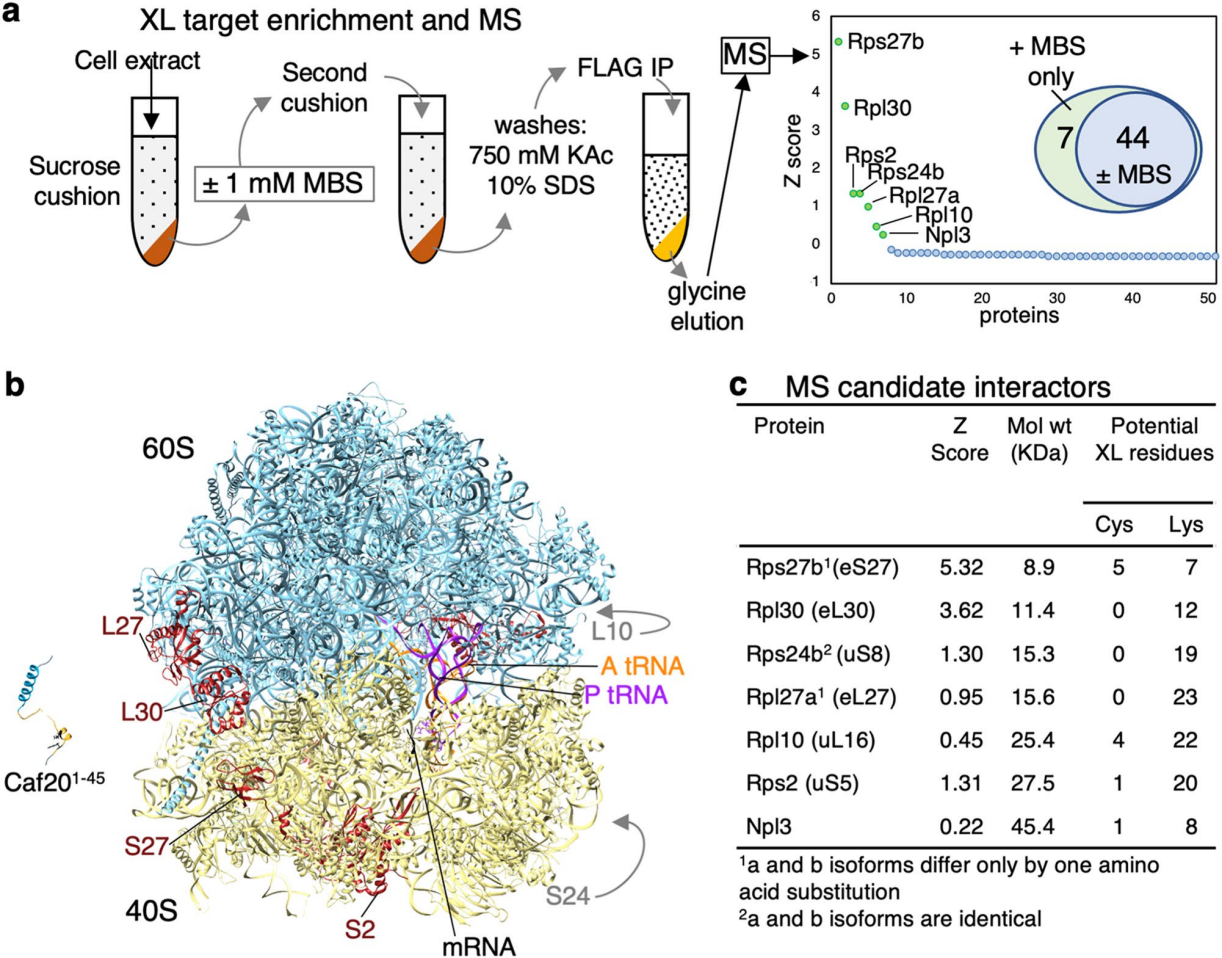
Crosslinking, purification and MS identifies candidate Caf20 interacting proteins. (**a**) XL and purification workflow overview and MS summary identifies 7 novel candidate Caf20 interacting proteins. (**b**) MS candidates placed on 80S ribosome (Pdb:6TNU)^19^, E site view, with Caf201-45 peptide (Pdb: 6FC3) as in Fig. 1 alongside to scale. Figure created with UCSF Chimera v1.15^39^ (**c**) properties of candidates found.

As previous attempts to identify Caf20-interacting proteins by mass spectrometry (MS) found several hundred candidates^15^, we combined MBS XL with successive sucrose cushion fractionations and stringent high salt and detergent washes before a final Flag immunoprecipitation and glycine elution of bound factors. The resulting purified samples were submitted to liquid chromatography MS/MS analysis. Four replicate samples along with two control samples where MBS was omitted were analysed in parallel. Seven MBS-specific proteins were identified and ranked by Z-score (Fig. 5a), six of which are ribosomal proteins (RP). Localising these subunits on an 80S ribosome electron cryo-microscopic reconstruction (cryoEM)^19^ revealed that four (Rpl27, Rpl30, Rps27 and Rps2 are located along a surface stripe spanning the two subunits (Fig. 5b), while Rps24 and Rpl10 are more remote (Fig. S7). The candidates with the highest Z score are Rpl30 and Rps27 which are closely positioned across the subunit division, as close as 12 Å, while Rpl30 and Rpl27 make direct contact with each other. All subunit molecular weights are broadly compatible with the major MBS XL species we identified (Fig. 5c).

The final Caf20-specific protein is Npl3, which is a multifunctional SR-like RNA-binding protein implicated in multiple steps in gene expression from transcription to splicing, nuclear export and translational regulation^20–22^. While all seven proteins were potentially of interest, at 45 KDa Npl3 is larger than our predicted major XL proteins and so our subsequent analyses focused on the ribosomal proteins.

### Synthetic genetic interactions between Caf20, Rps27B and Rpl27A

**Figure 6:**
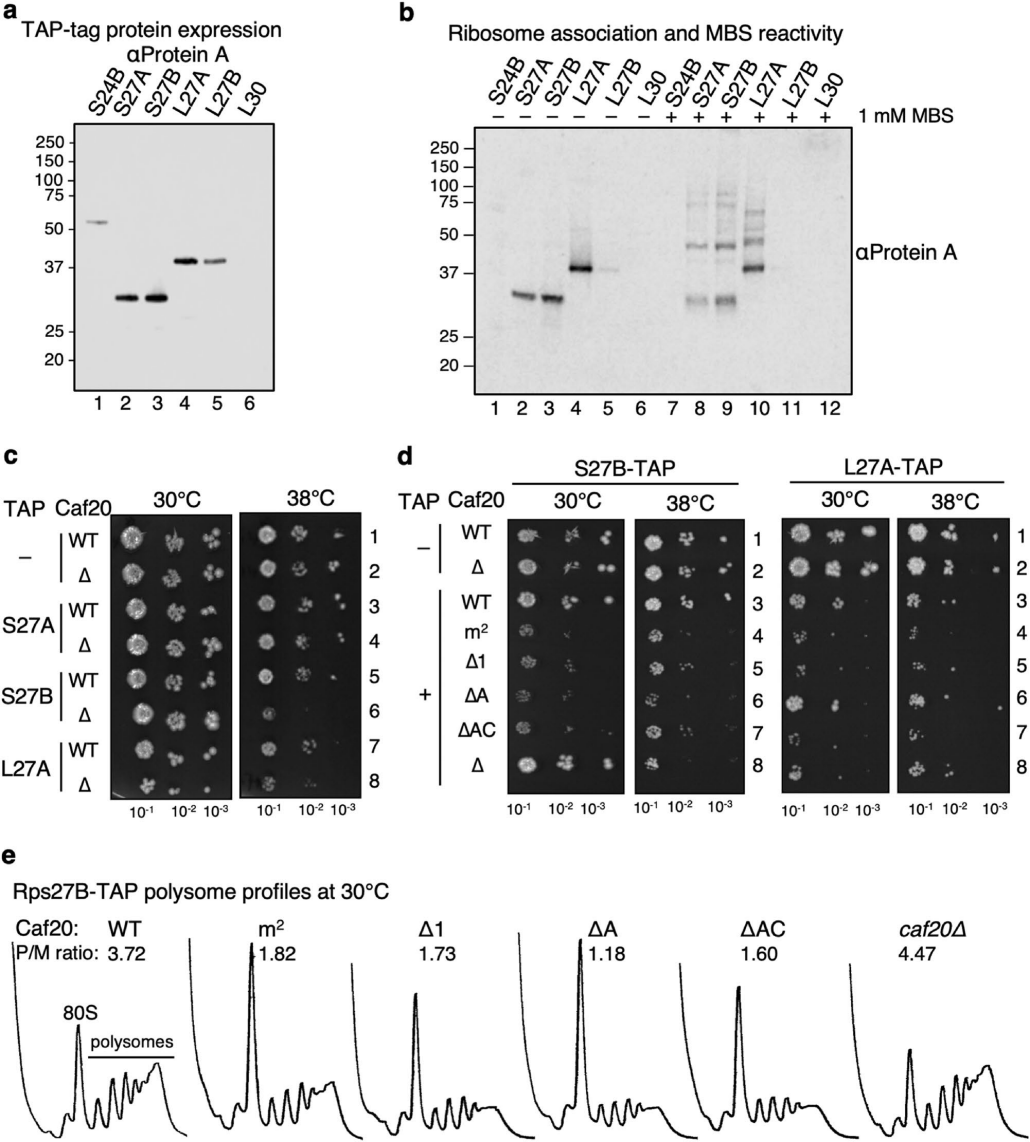
Genetic and biochemical validation of selected ribosomal protein targets. (**a**) Western blotting of total extracts of genomic TAP tagged ribosomal proteins probed for Protein A (TAP). (**b**) TAP tagged ribosomal proteins can be crosslinked with MBS. Sucrose cushion pellet fractions of TAP-tagged ribosomal protein strains ± 1 mM MBS. (**c**) Synthetic growth defect combining *caf20∆* with RPS27B-TAP and RPL27A-TAP strains. (**d**) Exacerbation of RPS27B-TAP *caf20*∆ and RPL27A-TAP *caf20∆* growth defects by expressing mutant Caf20 proteins. (**e**) Polysome profiles of Caf20 complemented *caf20∆* RPS27B-TAP strains during exponential growth at 30 °C in SC-leucine.

We were not able to source antibodies to these ribosomal proteins so instead investigated strains in our TAP-tagged collection. Three of these RPs are each paralog pairs which arose through a genome duplication event and the paralogs are either identical or differ by a single amino acid (Fig. 5c), making it unclear that the specific paralog designated by MS was correct, so we analysed both A and B isoforms, where possible. Rps24A, Rpl10 and Rps2 TAP strains were not available. This is likely because tagging many ribosomal proteins reduces cell fitness or is lethal. Of the strains tested only three (Rps27A, Rps27B and Rlp27A) expressed a protein of the expected size, associated well with ribosome pellets in sucrose cushion experiments and were cross-linked to other proteins with MBS (Fig. 6a,b) so we analysed these further.

We deleted Caf20 in these TAP strains by integrating a KanMX cassette at the Caf20 locus in each strain (Fig. S8a). The resulting strains grew well at standard temperature (30 °C), but rates of colony formation for both *RPS27B*-TAP *caf20*∆ and *RPL27A*-TAP *caf20∆* strains were reduced at 38 °C, indicating a synthetic temperature sensitive growth defect (Tsm^–^) was revealed (Fig. 6c, rows 6 and 8). In contrast no defect was seen with *RPS27A-TAP caf20∆* (Fig. 6c, row 4, left panels), similar to the lack of growth defect observed in the absence of any TAP-tag (Fig. S4a). To extend these analyses we transformed *RPS27B*-TAP *caf20*∆ strain with Caf20 plasmids used previously (Fig. S8b). As expected, introducing WT Caf20 suppressed the Tsm^–^ phenotype (Fig. 6d, row 3 versus 8). In contrast expressing mutants defective in eIF4E-interaction could not suppress the Tsm^–^ phenotype of *RPS27B*-TAP and even exacerbated the growth defect. In general, similar results were seen when the *RPL27A*-TAP strain combined with *caf20* mutants (Fig. 6d, right panels), although the WT rescue was partial. It seemed plausible that these TAP strains reduce ribosome function and that simultaneously altering Caf20 function combines to limit growth rates, consistent with both proteins functioning in the same pathway. To test this, we performed polysome profiling of the *CAF20*-transformed *RPS27B*-TAP strains shown in Fig. 6d following growth at 30 °C. This showed elevated 80S peaks and reduced polysome peaks for all four slow-growing Caf20 mutants, while the wild type and *caf20∆* profiles are typical of profiles for rapidly growing cells (Fig. 6e). The reduced polysome:monosome ratios are consistent with a translation initiation defect leading to the observed slow growth.

## Discussion

Caf20 shares the canonical 4E-BP motif, but unlike other 4E-BPs this motif is found directly at its amino terminus (Fig. S2). It was proposed following in vitro binding studies with a Caf20 N-terminal peptide that unlike other 4E-BPs, the NC helix does not contribute to the strength of the eIF4E interaction, despite forming part of the interface^9^. We therefore invistigated eIF4E-Caf20 interactions by a complementary approach combining mutagenesis with expression in yeast cells (Figs. 1 and 2) and extended the analysis through the entire protein sequence in a systematic way. The results of these experiments confirm that only the extreme N-terminal canonical motif is necessary for Caf20 to bind stably to eIF4E, even at 1 M salt. These results were obtained using lysates from optimally growing cells where translation is maximal. So as Caf20-eIF4E interations remain robust during active growth it suggests that Caf20 does not act entirely in the manner proposed for action of mammalian 4E-BP1/2. In mammals 4E-BP–eIF4E interactions are diminished during rapid growth, but promoted in quiescent cells.

In complementary experiments, we recently performed RNA IP and sequencing (Rip-Seq) to identify mRNAs enriched with the 5′ cap complexes. This revealed that mRNAs could be divided into distinct groups based on which proteins they preferentially enriched with. mRNAs preferentially associated with Caf20 were typically less-well engaged with ribosomes, and with longer ORFs^14^. Together the data suggest that Caf20 can contribute to the preferential engagement of mRNAs with the translation apparatus, but do not make it clear if or how Caf20 can be displaced by eIF4G to initiate translation, as Caf20-eIF4E interactions appear highly stable. In yeast eIF4G is less abundant than eIF4E, while Caf20 and eIF4E are approximately equimolar^23^. In contrast, in mammalian cells eIF4E is found at lower levels than eIF4G^24^. These ratio differences are likely important when considering how the relationships between eIF4E and its binding partners regulate protein synthesis in different organisms.

In contrast to eIF4E interactions, we found multiple Caf20 elements contribute to ribosome binding. The results are consistent with the idea that the amino and carboxy termini both contribute to ribosome binding. Caf20 is relatively rich in acidic residues and has an overall pI of 5.85, which is opposite to most ribosomal proteins which are basic. For example, the pIs of the six RP MS hits are between 9.35 and 11.2. Our results lead us to speculate that full length Caf20 may adopt an extended conformation lacking clear tertiary structure, as its amino terminus does (Figs. 1, 5) and as some other ribosome binding factors do, such as Stm1 and Lso2, which both bind inactive ‘hibernating’ ribosomes during stress^25,26^. However in contrast to these two factors which bind within the mRNA and tRNA-binding cavities, Caf20 appears to associate with polyribosomes in unstressed rapidly dividing cells where translation is maximal^15^. Hence it appears unlikely that Caf20 would interact with 80S ribosomes in a manner that competes with mRNA, tRNA or elongation factor binding.

The Caf20 interactor candidate proteins we identified were found across both 40S and 60S subunits and all have solvent exposed lysine residues that would be available for cross-linking with MBS (not shown). An extended conformation could enable Caf20 to bind in a position which affords cross-linking to more than one protein. However, it remains highly unlikely that all six RPs identified could be direct binding partners for such a small protein with a single cysteine residue. Rpl27 (eL27), Rpl30 (eL30) and Rps27 (eS27) are all closely located on the main body of the solvent surface away from the main functional centres for mRNA, tRNA and translation factor binding provides our best indication of a region on the surface where Caf20 might bind.

Rps27 has previously been implicated as contributing to eIF3 binding. eIF3 is a multisubunit translation initiation factor with several roles in translation including recruitment of the small ribosomal complex, scanning and AUG start codon recognition^4,27^. CryoEM of a partial yeast 48S preinitiation complex revealed that the multicomponent eIF3 complex was found bound on opposing sides of the 40S subunit^28,29^. The PCI domains of eIF3a and eIF3c sit on one side such that Rps27 is the main ribosome interaction contact for the eIF3c PCI domain. These eIF3 subunits have unfolded extensions that wrap around the 40S and the unresolved eIF3a extension was proposed to pass over another of our MS hits, Rps2 (uS5)^28,29^. As yeast eIF3c may interact with eIF4G by analogy with mammalian eIF3^2^, locating Caf20 close by could facilitate efficient exchange between eIF4E binding partners upon ribosome recruitment.

Elongating ribosomes can stall under a range of non-optimal conditions and the next ribosome coming up behind can collide with the stalled ribosome and form distinct stalled disome structures. One recent cryoEM structure of yeast ribosomes shows that contact is made between different segments of 18S rRNA of the 40S of the colliding ribosome with both Rps27 and Rpl27 of the leading, stalled ribosome^30^. However, there is no evidence that we are aware of, that Caf20 functions directly or indirectly to impact colliding ribosomes. eIF5A is a factor that is recruited to stalled, collided elongating ribosomes^31^ and mutants affecting eIF5A recruitment to ribosomes were shown to cause an increase in polysome:monosome ratios^32^. In contrast here Caf20 mutants caused reduced polysome:monosome ratios (Fig. 6e). This suggests Caf20-ribosome interactions are not assisting in resolving ribosome stalls, but does not rule out some other role.

Thus, we have demonstrated that Caf20 can interact with 80S ribosomes, but not whether it has a specific role when bound. It could simply be a passenger on the ribosome, or its position may facilitate rapid binding to available eIF4E upon dissociation of eIF4G from its complex with eIF4E. This idea is compatible with the previously proposed ‘cap-severed’ model for scanning where following recruitment of the 48S PIC to the 5′ cap, the initiation of scanning enables eIF4G to detach from eIF4E at the cap and to remain associated with the 48S PIC^4^. A local source of Caf20 could then rapidly bind to eIF4E and potentially antagonise other interactions of eIF4E. In this model mRNAs could be bound by eIF4G and Caf20 at the same time, despite their interaction with eIF4E being mutually exclusive. This idea is compatible with our prior RIp-Seq analysis which identified a subset of mRNAs with relatively long 5′UTRs that were enriched with eIF4E, eIF4G and both the yeast 4E-BPs^14^.

## Methods

### Strains, plasmids and growth conditions

Yeast strains used in this study are described in Table S1. Systematic deletion strains in BY4741 and BY4742 background^33^ while TAP-tagged His^+^ strains in the isogenic BY4741 background were sourced from Open Biosystems. 9xMyc and TAP tagged versions of Caf20 were made previously^34^. Strains made for this study used standard techniques^35^.

Plasmids are listed in Table S2. pAV2421 (also termed p*CAF20*-WT) [*CAF20*–FLAG_2_ *LEU2* 2µ] bears the *CAF20* wild type sequence and is C-terminally tagged with two copies of the Flag epitope^15^. pAV2422 (also termed p*caf20*^m2^) [*caf20*-Y4A-L9A-FLAG_2_ *LEU2* 2µ] expresses the Y4A, L9A double mutation in the core eIF4E-binding helix that disrupts eIF4E-binding^15,18^. These two plasmids were used as templates for site-directed mutagenesis (QuikChange, Agilent Technologies) to create different *caf20* mutant plasmid DNAs called ∆1-∆8 and ∆A-∆C with the help of synthetic oligonucleotide pairs listed in Table S3.

*CAF20* was deleted from the ribosomal protein TAP-tagged strains via transformation with the *caf20*-KanMX cassette amplified by PCR from GP4789 genomic DNA. Briefly, DNA was isolated with a genomic DNA purification kit (Promega), then PCR used the Phusion High-Fidelity DNA Polymerase (Thermo Scientific) and primers (CAF205′UTR F and CAF203′UTR R, Table S3). PCR products were concentrated with 3 M sodium acetate, pH 5.2 to a final concentration of 0.3 M sodium acetate and precipitated in ethanol. Approximately 15 µg of PCR product was used for deletion of *CAF20*. Deletion was verified by confirmation PCR and western blotting with Caf20 antiserum. Untagged and genomically integrated-tagged strains were grown at 30 °C on standard YPD and SCD complete 2% glucose media, while transformed strains were grown in SD minimal medium supplemented with auxotrophic supplements and on SCD–Leu (Flag-tagged plasmid studies) dropout medium^35^.

### Flag-tagged protein affinity purification and western blotting

Immunoprecipitation (IP) of Flag-tagged proteins was carried out with anti-Flag M2 magnetic beads as described^36^. See Table S4 for the key biochemical resource summary. For small-scale experiments, a 50–100 ml culture was grown at 30 °C to exponential phase of 0.6. The culture was then transferred into 50 ml Falcon tube and centrifuged at 5500×*g* for 5 min, 4 °C. The cells were washed in ice-cold IP lysis buffer (30 mM HEPES–KOH pH 7.5, 10% (w/v) glycerol, 1 mM TCEP-HCl pH 7.0) with salt concentration adjusted to either 100 mM or 1 M KCl and Pierce protease inhibitor tablet added just before use). Washed cells were pelleted at 5500×*g* for 5 min, 4 °C and resuspended in 1 ml and lysed with 500 µl of acid washed glass beads at 6 × 20 s. Extract was separated from beads and debris by centrifugation at maximum speed (16,100×*g*) for 4 min and transfer into a fresh tube before a second centrifugation at maximum speed for 20 min, 4 °C. 1 mg of the total cell extracts of each strain were diluted in 500 µl IP lysis buffer and mixed with 50 µl of Flag magnetic resin. This was incubated for 1–2 h on a tube rotator set at 30 rpm, 4 °C. The bound proteins were washed four times; two washes in IP lysis buffer and two washes in IP_low_ buffer (10 mM HEPES–KOH pH 7.5, 100 mM KCl, 10% (w/v) glycerol, 1 mM TCEP-HCl pH 7.0). The bound proteins were eluted from the beads in 50 µl of 200 µg/ml of 3X Flag peptide. SDS-PAGE and western blotting were performed using reagents indicated in Table S4.

Large cultures (2 L) were grown in flasks as above, harvested by centrifugation and lysed 3 × 2 min under liquid nitrogen using a 6870 Freezer Mill (SPEX SamplePrep). For large scale IP for mass spectrometry analysis, for every 1 g of cryogenic ground cell, 2 ml of IP lysis buffer was added. Once thawed on ice, tubes were centrifuged for 10 min at 5500×*g*, 4 °C. The lysate was further clarified by centrifugation in 5 ml Eppendorf tubes at 16,100×*g* for 20 min. The protein concentration was measured and processed accordingly. A pre-clearing step incubating extracts with Sepharose 4B agarose beads (Sigma) before Flag magnetic beads reduced non-specific binding.

### TAP-tagged protein affinity purification

TAP-magnetic beads, DYNAL Dynabeads Pan Mouse IgG, Monoclonal (Invitrogen) were used for TAP- affinity purification. The method was identical to the Flag IP described above until the final wash. The bound protein was then eluted from beads by boiling in 50 µl of 2X SDS sample buffer.

### MYC-tagged protein affinity purification

MYC-tagged protein purification was performed with MYC-agarose; EZview Red Anti-C-MYC Affinity Gel (Sigma). The buffer used was 1X LOLA (20 mM Tris–HCl, 140 mM NaCl, 1 mM MgCl, 10 U/ml RNAsin (Promega RNAsin Plus), phosphatase inhibitor cocktail 3 (Sigma), protease inhibitor cocktail (Pierce), 0.5% (v/v) NP40 (Igepal CA-420))^15^. The steps are equivalent to the Flag-tag purification described above but with small adjustments. The agarose beads were washed 5 times after IP. The bound protein on the resin was obtained by either boiling in 2X sample buffer or by eluting in low pH glycine buffer (0.2 M glycine, pH 2.6).

### Sucrose cushion for ribosome pelleting

Sucrose cushion fractionation was performed as described previously^15^. 50 ml of each yeast culture was grown at 30 °C to an OD_600_ of 0.6. Cycloheximide was added to the culture to a final concentration of 100 μg/ml, to trap translating ribosome-mRNA complexes. The culture was incubated for another 15 min at 30 °C, cells collected by centrifugation and lysed in chilled CSB buffer (300 mM Sorbitol, 20 mM HEPES pH 7.5, 1 mM EGTA, 5 mM MgCl_2_, 10 mM KCl, 10% (w/v) Glycerol, 100 μg/ml cycloheximide, 10 u/ml SUPERaseIn RNAse inhibitor and 1 Pierce protease inhibitor cocktail) with glass bead homogenization for 5 × 20 secs in low RNA-binding tubes (Life Technologies). 500 μg of the protein extract dissolved in 500 μl of CSB was gently lowered over a 400 μl of 50% (w/v) sucrose plus CSB buffer without sorbitol and protease inhibitor to form two distinct layers in Beckman thickwall polycarbonate tubes (Beckman). The tubes were centrifuged in an ultracentrifuge (rotor-TLA 120.2, used in Beckman Optima Max-XP centrifuge) for 2.5 h at 4 °C, 70,000 rpm. The position on the tube where the pellet was deposited was noted and the supernatant transferred into a new microfuge tube. The pellet was thoroughly resuspended in 100 μl Novex tricine SDS sample buffer (Invitrogen). To the supernatant fractions, TCA (trichloroacetic acid) was used to precipitate ribosome-free proteins. After acetone washes pellets were resuspended in 100 μl Novex tricine SDS sample buffer (supernatant fraction). 10 μl (10%) of both the pellet and supernatant fractions were analysed by SDS tricine gels and western blotting.

### SDS-PAGE and Western blotting analysis

Protein extracts were mixed with 2X SDS sample buffer and boiled at 95 °C for 10 min to denature protein complexes. Samples were resolved by SDS-or Tricine PAGE gels, transferred onto nitrocellulose membranes and probed with monoclonal and polyclonal antibodies listed in Table S4 and developed with Li-Cor secondary antibodies. Quantification used Li-Cor Image Studio software. Standard errors for three replicates were determined.

### Polysome profiling in sucrose density gradients

Polysome profiling was performed as described in^37^. 100 ml yeast cultures were grown at 30 °C to OD_600_ of 0.6. The yeast cells were harvested in cycloheximide to translating ribosomes running off from the mRNAs. Cycloheximide treatment (at a final concentration of 100 μg/ml) was performed in some Caf20 mutants that had reduced interactions with the ribosome and lysed in 200 μl polysome lysis buffer (20 mM HEPES pH 7.4, 2 mM magnesium acetate, 100 mM potassium acetate, 0.5 mM DTT and 100 μg/ml cycloheximide) for 20 s, 6–7 times. 2.5 OD units of the protein extracts were layered on sucrose gradients (15–50%) prepared in 12 ml polysome gradient thin-walled open polyallomer tubes (Seton Scientific) and separated with SW41 rotor set at 40,000 rpm, 2 °C for 2.5 h. The profile traces were fractionated with an ISCO-Brandel fractionator with absorbance (254 nm) recording. at and the images analysed with GNU Image Manipulation Program and ImageJ software^38^.

### Crosslinking of Caf20 to the yeast ribosome

In small scale experiments, 100 ml cultures were grown at 30 °C to an OD_600_ of 0.6. Cultures were treated with cycloheximide to a final concentration of 100 μg/ml for 15 min at 30 °C. The cells were processed for sucrose cushion, as described above. After ultracentrifugation, the pellet fraction was resuspended in CSB buffer (300 mM Sorbitol, 20 mM HEPES pH 7.5, 1 mM EGTA, 5 mM MgCl_2_, 10 mM KCl, 10% (w/v) glycerol, 100 μg/ml cycloheximide, 40 U/ml RNAse in (Promega) and 1 Pierce protease inhibitor cocktail tablet). This was then crosslinked with each of the three crosslinkers bismaleimidohexane (BMH), disuccinimidyl suberate (DSS) or m-maleimidobenzoyl-N-hydroxysuccinimide ester (MBS) at concentrations 0.5 mM, 1 mM and 2 mM at 30 °C for 20 min and quenched with stop solution (0.2 M DTT and 0.2 M ethanolamine). The crosslinked samples were processed for identification by western blotting. Where salt washes were included, the crosslinked samples were incubated with 500 mM or 750 mM potassium acetate added to the CBS buffer at 4 °C for 20 min. The high-salt treated extracts were centrifuged with the 50% (w/v) sucrose + CSB at 70,000 rpm, 4 °C for 2.5 h (Beckman rotor 120.2).

In larger scale crosslinking of ribosome-associated proteins, 3 L cultures per replicate were grown to an OD_600_ of 0.6 in SCD-leucine medium, cycloheximide (100 μg/ml) treated and harvested by centrifugation and liquid nitrogen grinding as described above. The protein concentration was diluted to 12 μg/μl before sucrose cushion. For the sucrose cushion, 4.9 ml of the protein extract was layered over 6.1 ml of 50% (w/v) sucrose + CSB in a 12 ml tube.

The sucrose cushion was performed twice in SW41 rotor set at 40,000 rpm, 2 °C for 3 h. After the first spin, the supernatant was transferred into another 12 ml dummy tube and centrifuge again. The two pellet fractions in the two dummy tubes were resuspended overnight in 150 μl CSB. All the pellets for each replicate were pooled together, then crosslinked with MBS to a final concentration of 1 mM MBS for 20 min and quenched with stop solution. The crosslinked samples were then treated with potassium acetate to a final concentration of 750 mM at 4 °C for 20 min. High-salt treated extracts were centrifuged with the 50% (w/v) sucrose + CSB at 40, 000 rpm, 0 °C for 12 h to separate ribosome-bound (pellet) and salt-labile cross-linked proteins(supernatant). The pellet was resuspended in SDS overnight at 0 °C in 500 μl of TE + 2% (w/v) SDS (20 mM Tris pH 7.6, 2 mM EDTA pH 8.0, Pierce protease inhibitor cocktail tablet and phosphatase inhibitor cocktail 3 (Sigma)). Ten ml of LOLA buffer without detergent (20 mM Tris–HCl, 140 mM NaCl, 1 mM MgCl, phosphatase inhibitor cocktail 3 (Sigma) and Pierce protease inhibitor cocktail tablet) was added to the SDS treated sample to bring down the concentration of the SDS to 0.1% (w/v) for IP. This was centrifuged at 40,000 rpm for 2.5 h. The supernatant was precleared with 100 μl washed Sepharose 4B agarose for 1 h at 4 °C and then was incubated with MYC agarose for 2 h at the same temperature.

After the IP, Myc-agarose beads were transferred to 2 ml Eppendorf tube with a wide pipette tip. The Myc agarose was washed five times in LOLA buffer without detergent at beads with spinning at 2000×*g* for 1 min, 0 °C. The bound proteins were eluted twice from the beads by incubating in 500 μl of 0.2 M glycine pH 2.6 for 1 h each. After each elution step, 500 μl of 1 M Tris (pH 8.0) was added to neutralize the pH and the beads were washed 3X with 150 μl of LOLA buffer minus detergent. The elutions and the washes were TCA precipitated and acetone washed and the pH pellet was normalized with 1 M Tris (pH 8.0). The pellet was dissolved and boiled in 50 μl SDS sample buffer. About 30 μl to 40 μl of the elution was loaded on a 12% (w/v) bis–tris, precast gel. The gel was stained with Instant blue stain (Expedeon Limited, Cambridge, UK) and the area of the bands of interest excised and submerged in water inside labelled low-protein binding tubes. The tubes were then sent for mass spectrometry protein identification at the University of Manchester, Faculty of Biology, Medicine and Health, PPMS for the Biological Mass Spectrometry Core Facility.

### Mass spectrometry identification by Label-free LC–MS/MS

Samples were dehydrated with acetonitrile and then centrifuged under vacuum. The dehydrated gels were reduced with 10 mM DTT and then alkylated with 55 mM iodoacetamide (IAM) by uniform modification of cysteine residues. The gel flakes were one after the other washed in 25 mM ammonium bicarbonate and then dried in acetonitrile. They were again washed and dried in ammonium bicarbonate and acetonitrile. After this, the gel flakes are vacuum centrifuged and then digested overnight with trypsin at 37 °C.

UltiMate 3000 Rapid Separation LC (RSLC, Dionex Corporation, Sunnyvale, CA) coupled to an Orbitrap Elite (Thermo Fisher Scientific) Mass Spectrometer was used to carry out label-free analysis of the trypsin-digested samples by LC–MS/MS. The peptides were concentrated on a pre-column (20 mm × 180 μm i.d., waters). The peptides were separated on a gradient, from 99% (w/v) A (0.1% (v/v) formic acid in water) and 1% (w/v) B (0.1% (v/v) formic acid in acetonitrile) to 25% (w/v) B, in 45 min at 200 nl/min, using 250 mm × 75 μm i.d. 1.7 mM BEH C18, analytical column (Waters). Peptides were selected from fragmentation automatically by data dependant analysis.

The MS data from the replicates were analysed on Scaffold 4.8.9 Software (www.proteomesoftware.com/products/scaffold/download) with 5 ppm peptide mass tolerance for the main search and 0.5 Da for the MS/MS fragment ions. The peak list was searched against the Uniprot *Saccharomyces cerevisiae* database from the built-in Andromeda search engine. The identified proteins were discarded when the peptides appeared in only one replicate. Identified proteins were analysed using standard tools available at Saccharomyces Genome Database, (www.yeastgenome.org). Enrichment was determined on a basis of a Z score [((XL/no-XL)—mean enrichment)/SD], Table S5 and Fig. 5.

## Supplementary Information

Supplementary Information.

## Data Availability

There are no restrictions to availability of materials and data presented in this work. All requests for information and materials should be sent to GDP.
