# Supplementary material for: Overlapping regions of Caf20 mediate its interactions with the mRNA-5′cap-binding protein eIF4E and with ribosomes
Source: Sci Rep. 2021 Jun 29;11:13467. doi: 10.1038/s41598-021-92931-4 (PMC8242001; doi:10.1038/s41598-021-92931-4)

Supplementary file for

Overlapping regions of Caf20 mediate its interactions with the mRNA-5'cap-binding protein eIF4E and with ribosomes

Ebelechukwu C. Nwokoye[1,2], Eiman AlNaseem[1], Robert A. Crawford[1], Lydia M. Castelli[1,3], Martin D. Jennings[1], Christopher J. Kershaw[1], Graham D. Pavitt[1,4].

Affiliations

1. Division of Molecular and Cellular Function, School of Biological Sciences, Faculty of Biology, Medicine and Health, Manchester Academic Health Science Centre, The University of Manchester, Manchester, M13 9PT, UK.

2. Department of Botany, Nnamdi Azikiwe University, Awka, Nigeria.

3. current address: Sheffield Institute for Translational Neuroscience (SITraN), Department of Neuroscience, University of Sheffield, Sheffield S10 2HQ, U.K.

4. Correspondence to Graham Pavitt: graham.pavitt@manchester.ac.uk

File contains:

Tables S1-S5

Figures S1-S8

Source data -uncropped western blot panels

Table S1. Yeast strains

Number	Genotype	Reference Source
BY4741	*MAT***a** *his3-Δ1 leu2Δ0 met15Δ0 ura3Δ0*	Ref 31
BY4742	*MATα his3Δ1 leu2Δ0 lys2Δ0 ura3Δ0*	Ref 31
GP4789	BY4742 *caf20Δ::KanMX4*	Ref 32
GP5094	BY4742 *CAF20-9xMYC::HIS3MX6*	Ref 32
GP5996	BY4742 *CAF20-TAP::HIS3MX6*	Ref 32
GP7833	BY4741 *RPS27A::TAP HIS3*	Open Biosystems
GP7834	BY4741 *RPS27B::TAP HIS3*	Open Biosystems
GP7835	BY4741 *RPL27A::TAP HIS3*	Open Biosystems
GP7838	BY4741 *RPS27A::TAP::HIS3 caf20Δ::KanMX4*	This study
GP7839	BY4741 *RPS27B::TAP HIS3 caf20Δ::KanMX4*	This study
GP7840	BY4741*RPL27A::TAP HIS3 caf20Δ::KanMX4*	This study

Table S2. Plasmids

Number	Genotype	Desig-nation	Reference/ Source
pAV2421	*CAF20-FLAG₂ LEU2*	WT	Ref 15
pAV2422	*caf20^m2^-FLAG₂ LEU2*	m2	Ref 15
pAV2475	*caf20Δ3-22-FLAG2 LEU2*	Δ1	This study
pAV2478	*caf20Δ23-42-FLAG2 LEU2*	Δ2	This study
pAV2479	*caf20Δ43-62-FLAG2 LEU2*	Δ3	This study
pAV2482	*caf20Δ63-82-FLAG2 LEU2*	Δ4	This study
pAV2484	*caf20Δ83-102-FLAG2 LEU2*	Δ5	This study
pAV2488	*caf20Δ103-122-FLAG2 LEU2*	Δ6	This study
pAV2491	*caf20Δ123-142-FLAG2 LEU2*	Δ7	This study
pAV2493	*caf20Δ143-161-FLAG2 LEU2*	Δ8	This study
pAV1302	pRS425 *LEU2*	V	
pAV2525	*caf20*Δ3-48-FLAG2 *LEU2*	ΔA	This study
pAV2527	*caf20*Δ49-107-FLAG2 *LEU2*	ΔB	This study
pAV2535	*caf20*^{m2} Δ49-107-FLAG2 *LEU2*	ΔB^{m2}	This study
pAV2528	*caf20*Δ108-161-FLAG2 *LEU2*	ΔC	This study
pAV2527	*caf20*Δ49-107-FLAG2 *LEU2*	ΔB	This study
pAV2535	*caf20*^{m2}Δ49-107-FLAG2 *LEU2*	ΔB^{m2}	This study
pAV2528	*caf20*Δ108-161-FLAG2 *LEU2*	ΔC	This study
pAV2536	*caf20*^{m2}Δ108-161-FLAG2 *LEU2*	ΔC^{m2}	This study
pAV2530	*caf20*Δ3-107-FLAG2 *LEU2*	ΔAB	This study
pAV2532	*caf20*Δ3-48, 108-161-FLAG2 *LEU2*	ΔAC	This study
pAV2533	*caf20*Δ49-161-FLAG2 *LEU2*	ΔBC	This study
pAV2538	*caf20*^{m2}Δ49-161-FLAG2 *LEU2*	ΔBC^{m2}	This study

Table S3. Oligonucleotide primers

Name	Nucleotide sequence (5' to 3')
CAF20Δ1F	CACGACATGATCGATGCGGTGGAATTTAGAGCCATC
CAF20Δ1R	TTCCACCGCATCGATCATGTCGTGAAATTAAATAAAAG
CAF20Δ2F	GGAAGTTAATTTCGAAGAGTTTAACAGTCATCATGTTGG
CAF20Δ2R	GTTAAACTCTTCGAAATTAACTTCCAAAGTTAAACTTGG
CAF20Δ3F	CAACACTTGAAAGAGGGTAGACCAAAGATTAAGCACAAC
CAF20Δ3R	CTTTGGTCTACCCTCTTTCAAGTGTTGCAATTGCTTAAC
CAF20Δ4F	CTTCCCACCATCATACATTTGAAGCCAAGAAGAAGGG
CAF20Δ4R	GGCTTCAAATGTATGATGGTGGGAAGATCTTCTACGACC
CAF20Δ5F	CAGATGGTTGGTGCCCAACTTCTACTGTGCCAGTTGCTAC
CAF20Δ5R	CACAGTAGAAGTTGGGCACCAACCATCTGAATCGGTTGTAAC
CAF20Δ6F	GAAACAGAAACCACAAACAAAAATATTTCTTCCAACAGACC
CAF20Δ6R	GAAATATTTTTGTTTGTGGTTTCTGTTTCTTCTTCATC
CAF20Δ7F	GTCAAGCCAAATATTCTTGGTTTCAACGCATTTGCTGC
CAF20Δ7R	GAAACCAAGAATATTTGGCTTGACTTTTAAAGTTTC
CAF20Δ8F	GCGGACAAGCCATCCGGAGACTACAAGGACGACGATGAC
CAF20Δ8R	GTAGTCTCCGGATGGCTTGTCCGCAACAATATCTCTGG
CAF20A F	CACGACATGATCCATGTTGGTCATTTCGGTCGTAGAAG
CAF20A R	CCGAAATGACCAACATGGATCATGTCGTGAAATTAAATAAAAG
CAF20B F	GAGTTTAACAGTCATCCAGTTGCTACCATTGCCCAAGAAAC
CAF20B R	GGGCAATGGTAGCAACTGGATGACTGTTAAACTCTTCCTC
CAF20C F	CAACTTCTACTGTGTCCGGAGACTACAAGGACGACGATG
CAF20C R	GTAGTCTCCGGACACAGTAGAAGTTGGTGTGGTTTCTG
CAF20BC F	GAGTTTAACAGTCATTCCGGAGACTACAAGGACGACGATGAC
CAF20BC R	GTAGTCTCCGGAATGACTGTTAAACTCTTCCTCTTTCAAGTG
CAF20AB F	CACGACATGATCCCAGTTGCTACCATTGCCCAAGAAAC
CAF20AB R	GGGCAATGGTAGCAACTGGGATCATGTCGTGAAATTAAATAAAAG
CAF20A2 F	CATGTTGGTCATTTCGGTCGTAGAAG
CAF20A2 R	GATCATGTCGTGAAATTAAATAAAAGTTCTTAATCC
CAF20AB2 F	CCAGTTGCTACCATTGCCCAAG
CAF20AB2 R	GATCATGTCGTGAAATTAAATAAAAGTTCTTAATCC
CAF205'UTR F	CATTATTTGAGCTGTAACCTGAATATAGG
CAF203'UTR R	GAGTAAAAACTGTTTATTAAAAAAAATGTTATTCA

Table S4. Key biochemical resource summary

Resource	Source	Specification
**Antibody**		
Rabbit Anti-FLAG	F7425, Sigma	1: 1000
Mouse Anti-FLAG	F1804, Sigma	1: 1000
Chicken anti-ECS (DDDDK)	Part: A190-100A, Universal Biologicals (Cambridge)	1: 2000
Rabbit Anti-eIF4E	Mark Ashe	1: 5000
Rabbit Anti-Caf20	Mark Ashe	1: 5000
Rabbit Anti-eIF4G	Mark Ashe	1: 5000
Rabbit Anti-Rpl35	Martin Pool	1: 5000
Rabbit Anti-Rps3	Martin Pool	1: 50,000
Mouse Anti Pgk1	[22C5D8] Abcam ab113687	1:10,000
Polyclonal anti-rabbit/ anti-mouse/ anti-chicken	Lot# C60321-05; Lot# C60107-03; Lot# C41029-14; Li-Cor	1:10,000
Polyclonal anti-chicken	Goat anti-Chicken IgY, Invitrogen	1:10,000
IRDye® 800CW Protein Labelling Kit	Part number: 928-38044, Li-Cor	1.71 µl dye to 1 mg of antibody
**Affinity Resins**		
Anti-FLAG (R) M2 magnetic beads	M8823-5ML, Sigma	50 - 100 µl / sample
TAP-magnetic beads, *DYNAL Dynabeads Pan Mouse IgG, Monoclonal*	11041, Invitrogen	50 µl / sample
MYC-agarose; EZview Red Anti-C-MYC Affinity Gel	Part number: E6654, Sigma	50 µl or 100 µl / sample
3X FLAG peptide	F4799, Sigma	200 µg/ml
Sepharose® 4B agarose (45-165 µm)	4B200, Sigma	100 µl/ sample
**Equipment**		
Beckman thickwall polycarbonate tubes	Part no. 343778, Beckman	
Lo bind, RNase-free Microfuge Tubes	AM12450, Life Technologies	1.5 ml tubes
**Reagents**		
Promega Wizard Genomic DNA purification kit	Cat No: A1125, Promega	
Phusion® High-Fidelity DNA Polymerase	Catalog No: M0530S, New England BioLabs	
G418 disulfate salt	A1720, Sigma	200 µg/ml
Cycloheximide	A0879.0005, VWR	100 µg/ml
Pierce protease inhibitor, EDTA-free tablets	A32965, ThermoFisher Scientific	1 tablet/50 ml buffer
Phosphatase inhibitor cocktail 3	P0044, Sigma	1% (v/v)
RNAsin Plus	N2611, Promega	40U and 10U/ml
SUPERase In RNAse inhibitor	AM2694, Life Technologies	10 u/ml
Novex tricine SDS sample buffer	LC1676, Invitrogen	
Crosslinkers:<br>BMH	22330, ThermoFisher Scientific	0.5, 1 and 2 mM
DSS	21655, ThermoFisher Scientific	0.5, 1 and 2 mM
MBS	22311, ThermoFisher Scientific	0.5, 1 and 2 mM

Table S5. Table of Mass Spectrometry identified proteins of ribosome extract crosslinking

Identified Proteins	MW (KDa)	XL*				No XL*		Z score
		A	B	C	D	E	F	
RPS27B	8.9	2	2	4	2			5.32
RPL30	11.4	4	2	3				3.62
RPS2	27.5	5		4				1.31
RPS24B	15.3	2		3				1.30
RPL27A	15.6	2		2				0.95
RPL10	25.4	2		2				0.45
NPL3	45.4			2	3			0.22
RPS13	17.0	5	3	5			2	-0.15
RPS17B	15.8	3	2	5			3	-0.23
RPS20	13.9	2	2	2	2		3	-0.24
RPL1A	24.5	2	3	4			2	-0.25
RPL28	16.7	3		3		2		-0.25
RPS18B	17.0	3	3	3			3	-0.25
RPL17B	20.6	3	2	2			2	-0.25
RPL14B	15.2	3		3			3	-0.27
RPL20B	20.5	5	2			3		-0.28
RPS1B	28.8	3	2	3			3	-0.29
RPL13A	22.6	2		4			3	-0.29
RPS1A	28.8	6	2	7			6	-0.29
STM1	30.0		2	3			2	-0.30
RPL7A	27.7	4		5			4	-0.30
RPS5	25.0	3	4	5	2	2	5	-0.30
RPS3	26.5	4	8	9			10	-0.30
RPS9A	22.5	3		4			4	-0.30
RPL8B	28.1	6	2	9	2	2	7	-0.30
RPL6B	20.0	4	2	3		2	4	-0.30
RPS6A	27.0	5		3		4		-0.30
RPL23B	14.5	2		2			4	-0.30
ADH1	36.8	2		4			3	-0.31
RPS0B	27.9	2	3	5	3	3	6	-0.31
RPL4A	39.1	8	5	10	2	4	10	-0.32
RPL12A	17.8			2	2		5	-0.32
RPS8B	22.5	3		2		5		-0.32
NMD3	59.1	3		8	2		5	-0.32
TEF1	50.0	4	8	8	3	3	9	-0.32
RPP0	33.7			3	2		4	-0.32
ASC1	34.8	3	4	9			13	-0.32
RPL15B	24.5	4		2		3	4	-0.32
DBP2	61.0	2	3	13			9	-0.32
TY1	49.1	2	2	4			5	-0.32
RPL3	43.8	11		8		8	8	-0.32
SSB2	66.6	7	14	18	17	4	29	-0.33
SSZ1	58.2		8	8	8		20	-0.33
GCD11	57.9	2	4	6	2		12	-0.33
UTP15	57.7		2	7			8	-0.33
SSA2	69.4		2		4		5	-0.33
ZUO1	49.0	2	2	6	2	2	13	-0.33
NOP56	56.9	3	3	4		2	11	-0.33
PDC1	61.5			5	2		9	-0.33
NOP58	57.0	4		4		4	8	-0.33
CDC19	54.5			10	3		21	-0.33

*Number of peptides identified in each replicate A-F

Supplementary Figure Legends

Figure S1. Loading Control for Figure 1.

Coomassie stained gel of total-protein cell lysates.

Figure S2. Caf20 Clustal alignment and secondary structure predictions.

a) multiple sequence alignment of Caf20 related sequences performed with the Clustal Omega server. The conserved canonical eIF4E binding site residues mutated in m^2 are in bold and the positions of the Δ1-Δ8 20 residue consecutive deletions are denoted. Larger regions A-C each comprise a single aligned row. b) alignment based secondary structure predicted elements and the same found in crystal structure 6FC3[9]. Alpha helices are marked as red tubes, and beta strands as green arrows. Canonical (C) and non-canonical (NC) helices shown to bind eIF4E are marked.

Figure S3. Relative expression levels of Caf20 Mutants**.**

Left, Cartoons showing deletions made. Right, western blots of total protein extracts of yeast strain GP4789 (Supplementary Table 1) bearing indicated Caf20 mutant or WT on a plasmid as the sole source of Caf20 or a vector control (lanes 11).

a) 20 residue deletions Δ1-Δ8 and controls. b) Larger deletions. Quantification of signal (LI-COR Odyssey Fc) relative to eIF4E levels ± standard deviation, n=3 (a) or Pgk1 (b).

Figure S4. Cell growth and polysome profiling reveals no major impact of mutations unstressed cells. a) Serial dilution growth assays for strains at 30°C and 38°C on SC-leucine medium for 2 days b) Polysome to monosome ratios (P/M) estimated from GIMP image program are shown. The 80S peaks in $Caf20^{m2}$, $Caf20^{\Delta AC}$, $Caf20^{\Delta A}$ and $Caf20^{\Delta AB}$ appear slightly higher than the Caf20 and $Caf20^{\Delta C}$ but does not significantly affect the P/M ratio. n=3.

Figure S5. No evidence for Caf20 homodimer formation in vivo.

a) Cartoon overview of use of 2 tagged versions of Caf20 (9xMyc chromosomal copy and 2xFLAG on a plasmid) to evaluate dimer formation during FLAG IP. b) Myc-Caf20-Myc does not co-purify with Caf20-FLAG constructs on FLAG M2 affinity resin. Western blotting of total cell extracts and FLAG peptide eluted purifications from Caf20-9MYC-tag strain (GP5094) transformed with indicated *CAF20-FLAG* plasmids or an empty vector (–). n =1.

Supplementary Figure S6. Further characterising Caf20 crosslinking reactions.

a) Major MBS crosslinked bands are independent of the epitope tag, but not all cross-linked partners can be equally enriched by affinity purification. b, c) MBS and d) BMH crosslinks are dependent on cysteine 82, lost in Caf20$^{\Delta 4}$, but retained when XL to Caf20^{m2} and Caf20$^{\Delta AC}$.

Supplementary Figure S7. Alternative ribosome view.

MS candidates placed on 80S ribosome (Pdb:6TNU), as in Fig.5 but rotated view to indicate Rps24 (uS8) and Rpl10 (uL16). Figure created with UCSF Chimera v1.15[39].

Supplementary Figure S8. Western blotting validation of RP-TAP strains.

a) Confirmation of deletion of *CAF20* in RP-TAP strains. b) Confirmation of mutant expression. Lane 3 is a replicate sample superimposed here as original candidate clone had no flag signal and was replaced.

Figure S1.

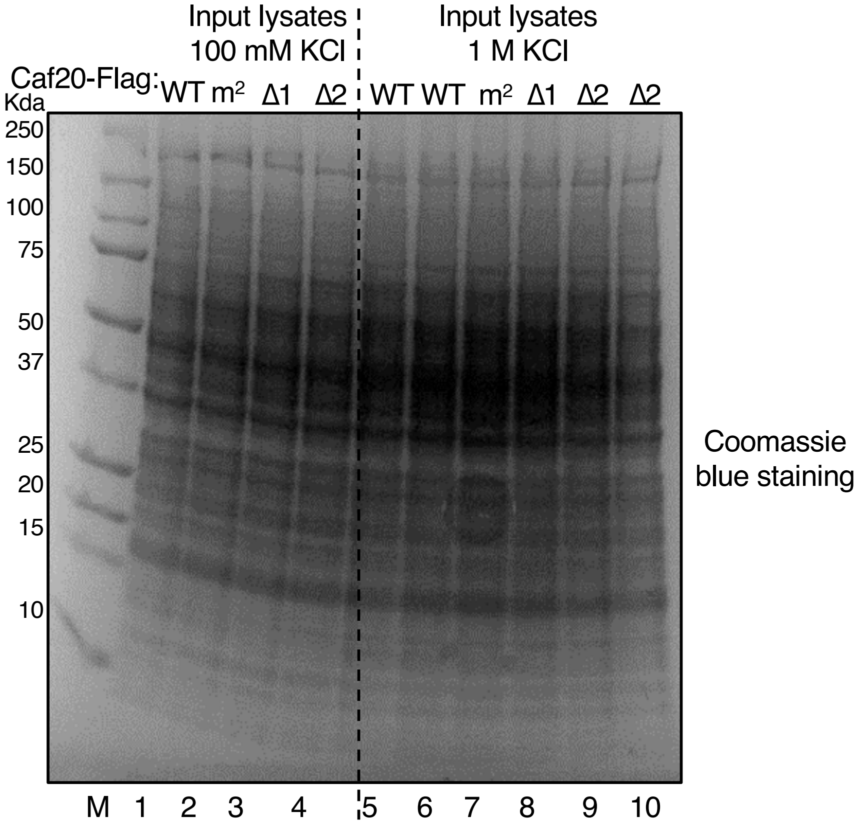
Input lysates
100 mM KCl
Input lysates
1 M KCl
Caf20-Flag:
WT m2 Δ1 Δ2 WT WT m2 Δ1 Δ2 Δ2
Kda
250
150
100
75
50
37
25
20
15
10
Coomassie
blue staining
M 1 2 3 4 5 6 7 8 9 10

Figure S2

a

```
CLUSTAL O(1.2.4) Caf20 homolog multiple sequence alignment

                    4    9           °                      °
E.gossypii          MAKYTEEELLQLKPTYDVAVNFDVDAFKAMIAEVAEHHEIADL-F----------------HQ-    44
K.lactis            MIRYTEEELLQLRPTEPVKPNFDVDEFNAIIEKVKEIQEAHEEEF----------------SH-    45
C.glabrata          MIKYSIDELIQLKPSETLKVSFDHVEFRNIIEKVVELQKLKEEEF----------------HSH    48
S.cerevisiae        MIKYTIDELFQLKPSLTLEVNFDAVEFRAIIEKVKQLQHLKEEEF----------------NSH    48
V.polyspora         MVRYTVDELFHLKPTESLPVQFDAEEFKAIIEKVKQIQALKEEEF----------------NAH    48
M.guilliermondii    MVKYTEDELLEFQEIAYNPQPQVLDAFNQMVDEVREHANAEIERQKHLKWSNGDTYIDENGNER    64
C.albicans          MAKYTEEQLLELKSEAHTPKPEILDAFNKLIEEVKESIE--QHQQHQRKWHNGDTYIDEHGHER    62
                    * :*: ::*:.::              *. :: :* :
                      Δ1                    Δ2                    Δ3

                              °                      °                      °
E.gossypii          ----KARRRSSHHHGV--KPKIKA-HKPRITTDDDGWCTSTRKTSVV-AVGDDGE----PSPAF    98
K.lactis            ------FRRRSSHHHA--KPKFKH-LKPKITTDEEGWSTLETAPAVR-RKSVA---EEEEPTIV    98
C.glabrata          H----GNRRRSSHHHM--KPKIKH-NKPKVKTDADGWSTLEPATAGH-EEESSSSAT-PAAAAT    103
S.cerevisiae        HVGHFGRR-RSSHHHG--RPKIKH-NKPKVTTDSDGWCTFEAKKKGS-GEDDEEETETTPTSTV    107
V.polyspora         G-GHFNRRRSSHHHHG--RPKVKH-TKPKVTTDSDGWSTFEAANKKV-NEDEESENV-------    100
M.guilliermondii    PYHHLNRRRGSRSG---AKPNLKRKGAESVTVDDDGWATLAKPKKS-FGAEEAGEERTKF----    120
C.albicans          SYHHINRRRQSKGASGVPRPNLRKK--SEPVVDEDGWATLSKPKKGSFAEGDAIEERIKF----    120
                           *  *        :*:.:         .* :**.*
                                          Δ4                   Δ5

                              °                     °                     °
E.gossypii          VAQETLRVKPNNKNIASSRPADTRDIVADKPTMSFNAFAALESDDEDQF-------   147
K.lactis            IAQETLKVKPN-KHISSSRPADARDIVADKPSKAFNAFAALESDEEEEQE------   147
C.glabrata          TKTGAPQETIRVKPNNKNISSSRPADNSDIIADKQTHGFNAFAALEDEEDE-----   154
S.cerevisiae        PVATIAQETLKVKPNNKNISSNRPADTRDIVADKPILGFNAFAALESEDEDDEA--   161
V.polyspora         -SVAVVPETLKVKPNNKNISSSRPADNKDIIADKQTHSFNAFAALESDEEEET---   152
M.guilliermondii    RDSLKD-GSVKARPNNKNLGSSKAVDPRDAIADKNTISFNAFEALGDDSDDDDE--   173
C.albicans          RETNNSGAGIKARPNNKNLGSSKAVDPREIASDKQTKAFNAFAALGDEDDDDEDDE   176
                                ::.:** *::.*.: .*  :  :**   .**** ** .:.::
                       Δ6                  Δ7                   Δ8
```

(In S.cerevisiae, the C in "SDGWCTFEAKKKGS" is printed bold and underlined.)

NP_014919 Saccharomyces cerevisiae S288C
XP_452523 Kluyveromyces lactis
NP_985201 Eremothecium gossypii
A5DEY7 Meyerozyma guilliermondii
A7TRH1 Vanderwaltozyma polyspora
Q6FKJ9 Candida glabrata
Q5AQ12 Candida albicans

b

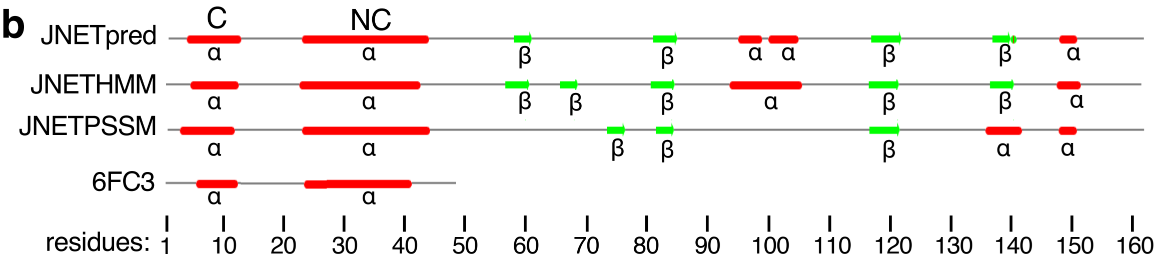

Figure S3

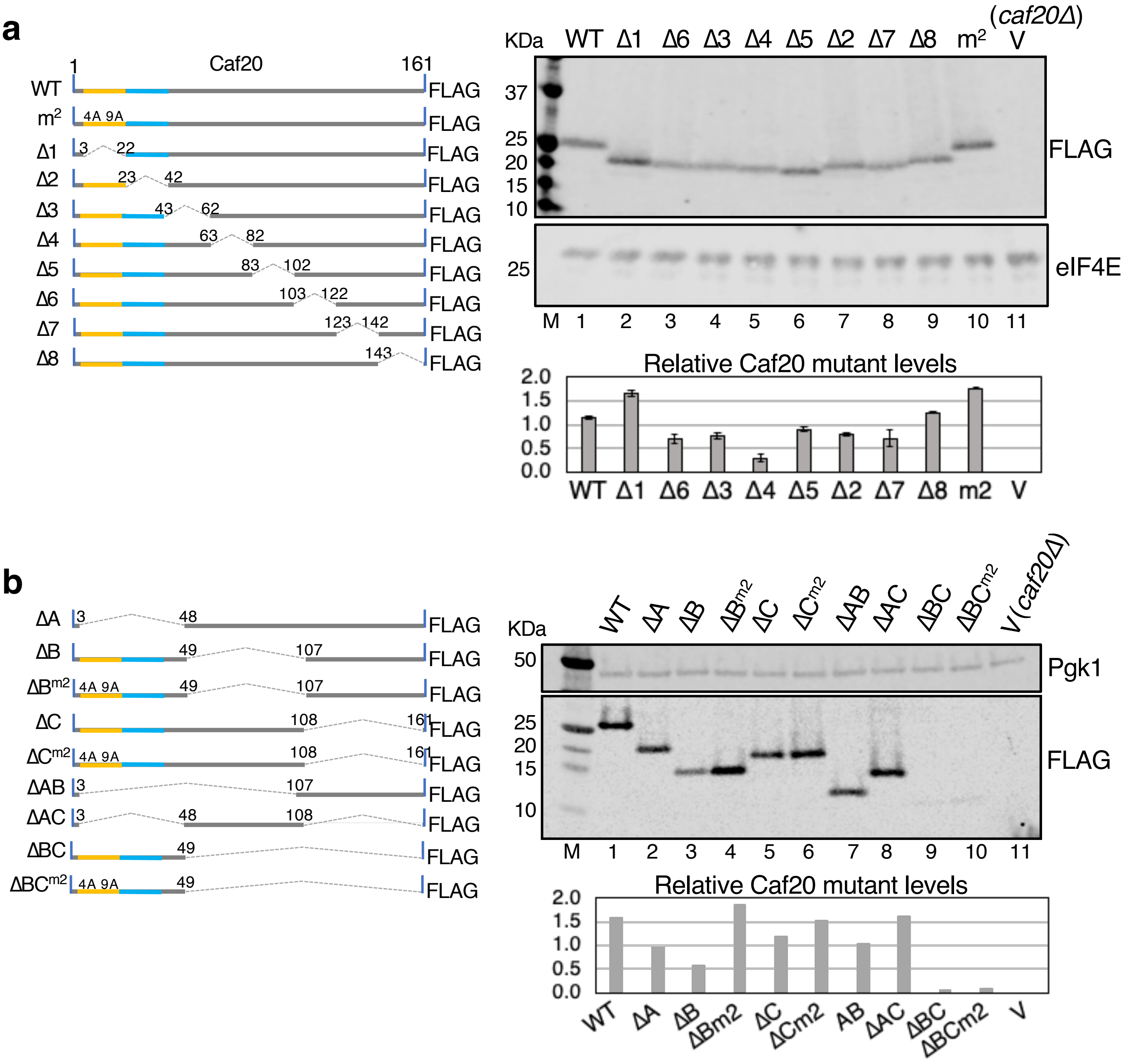
a
Caf20
1
161
WT
m2
4A 9A
Δ1
Δ2
Δ3
Δ4
Δ5
Δ6
Δ7
Δ8
FLAG
(caf20Δ)
KDa
FLAG
eIF4E
M 1 2 3 4 5 6 7 8 9 10 11
Relative Caf20 mutant levels
WT Δ1 Δ6 Δ3 Δ4 Δ5 Δ2 Δ7 Δ8 m2 V
b
ΔA
ΔB
ΔBm2
ΔC
ΔCm2
ΔAB
ΔAC
ΔBC
ΔBCm2
V(caf20Δ)
Pgk1
FLAG
Relative Caf20 mutant levels

Figure S4

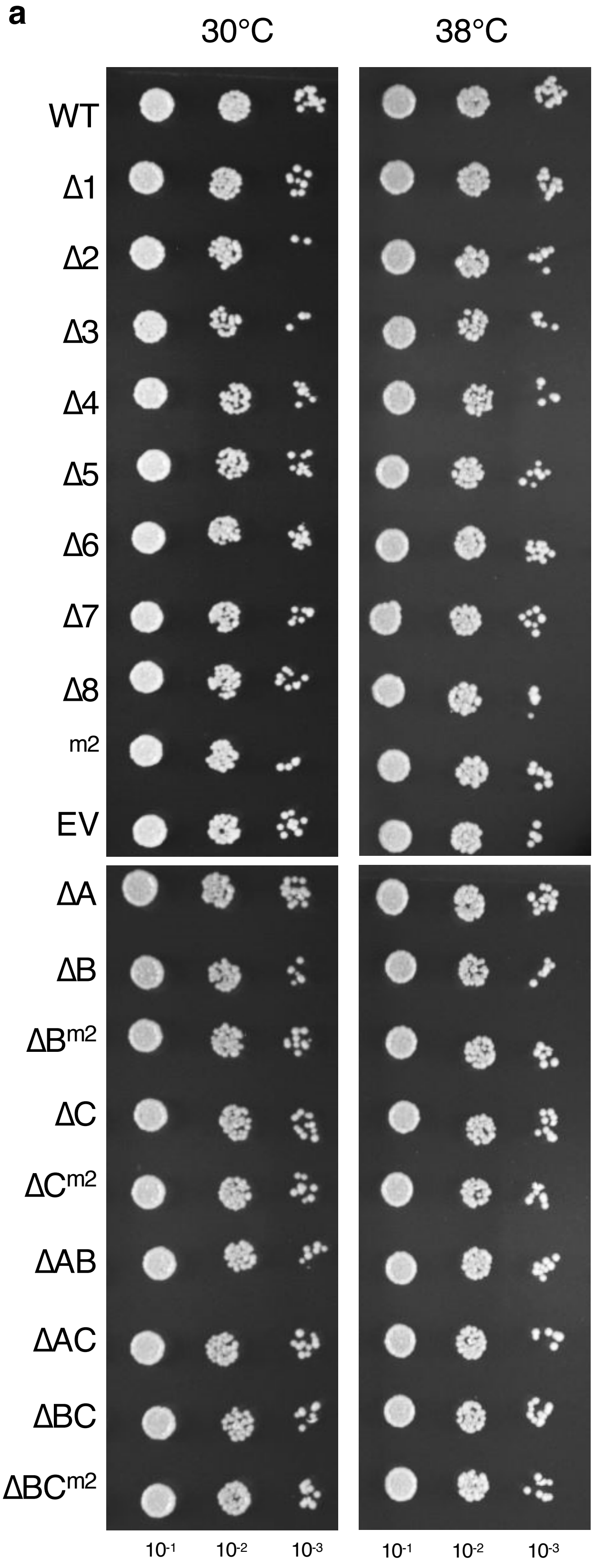
a
30°C
38°C
WT
Δ1
Δ2
Δ3
Δ4
Δ5
Δ6
Δ7
Δ8
m2
EV
ΔA
ΔB
ΔB m2
ΔC
ΔC m2
ΔAB
ΔAC
ΔBC
ΔBC m2
10-1 10-2 10-3 10-1 10-2 10-3

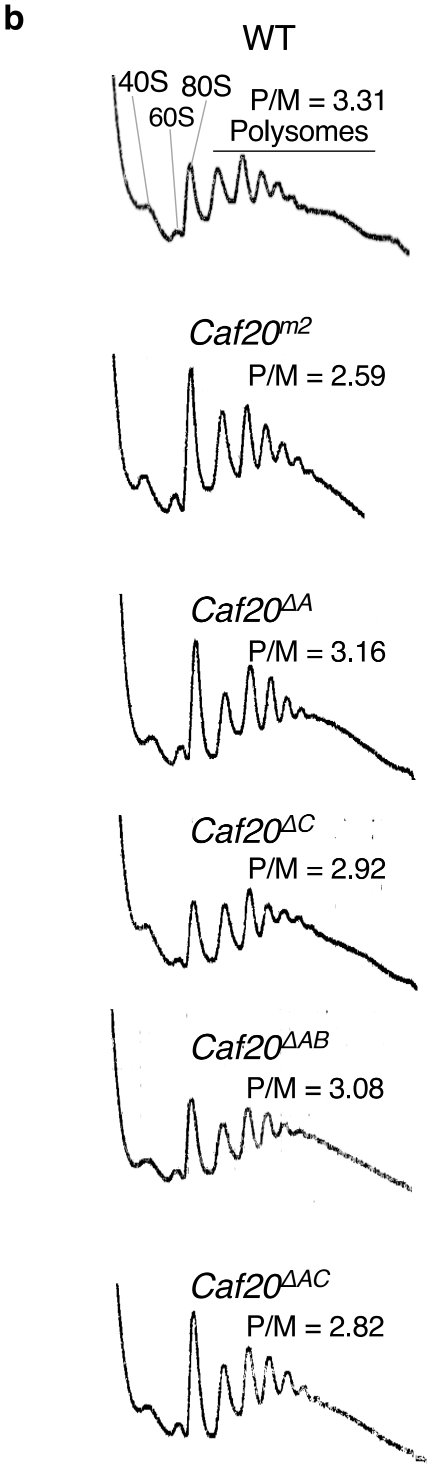
b
WT
40S
80S
60S
P/M = 3.31
Polysomes
Caf20 m2
P/M = 2.59
Caf20 ΔA
P/M = 3.16
Caf20 ΔC
P/M = 2.92
Caf20 ΔAB
P/M = 3.08
Caf20 ΔAC
P/M = 2.82

Figure S5

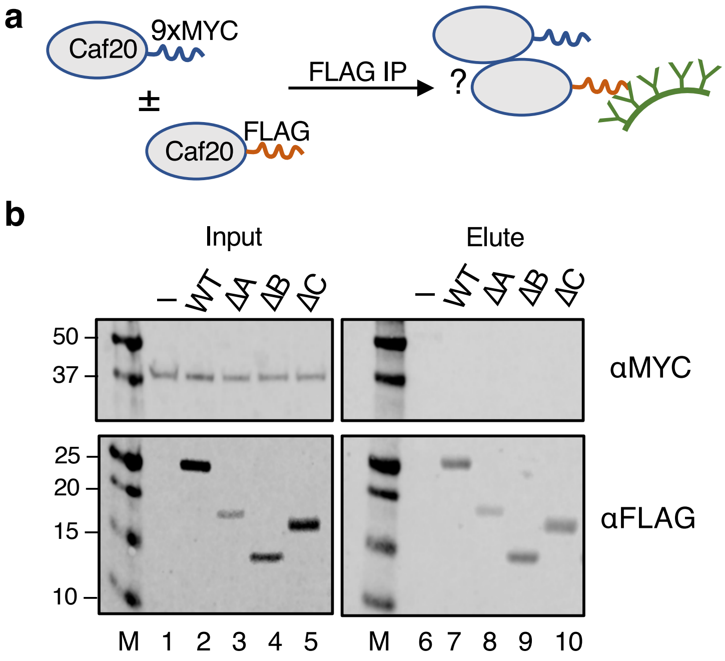
a
Caf20
9xMYC
±
Caf20
FLAG
FLAG IP
?
b
Input
Elute
– WT ΔA ΔB ΔC
– WT ΔA ΔB ΔC
50
37
25
20
15
10
αMYC
αFLAG
M 1 2 3 4 5
M 6 7 8 9 10

Figure S6

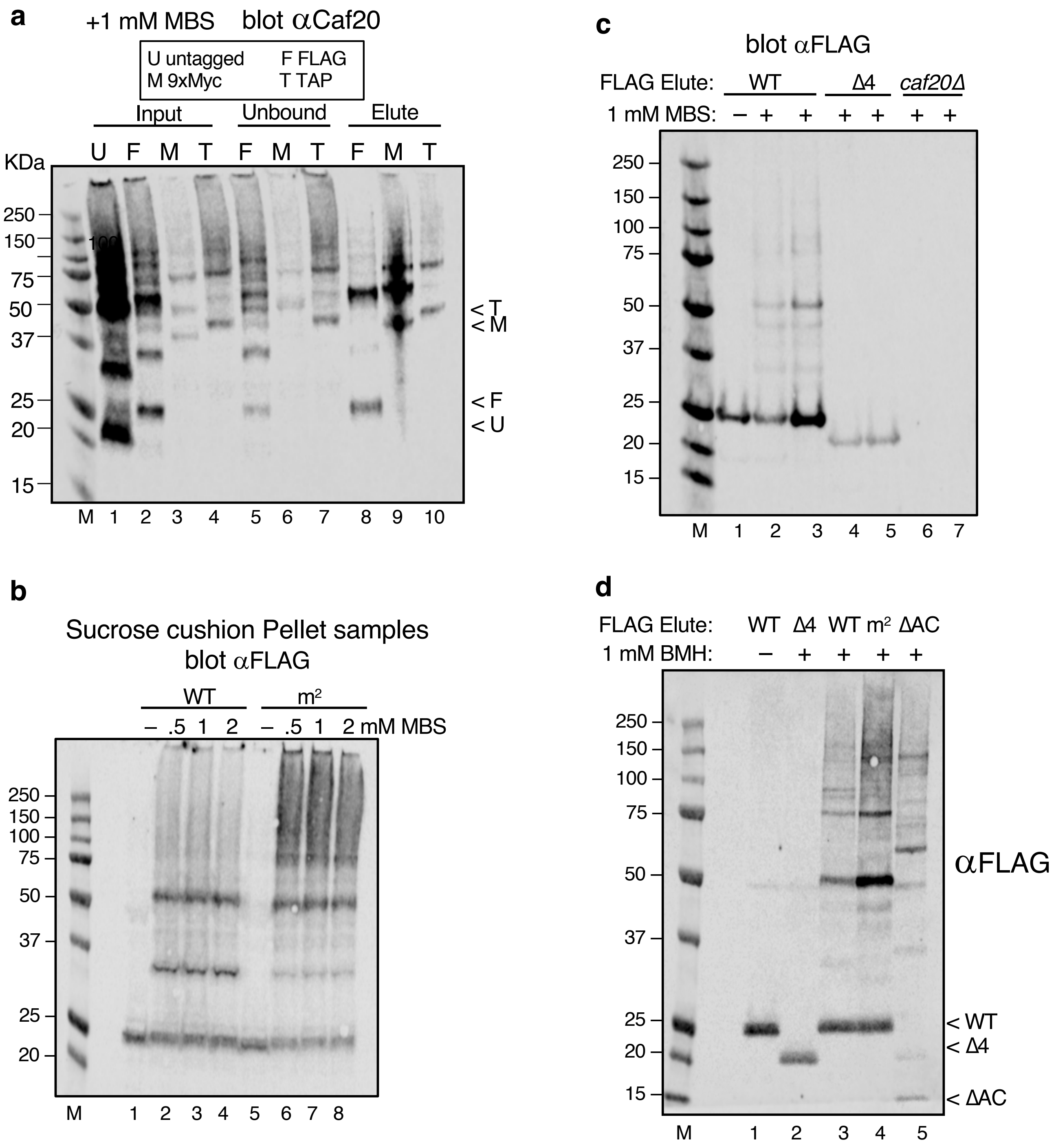
a
+1 mM MBS blot αCaf20
U untagged F FLAG
M 9xMyc T TAP
Input Unbound Elute
U F M T F M T F M T
KDa
250
150
75
50
37
25
20
15
< T
< M
< F
< U
M 1 2 3 4 5 6 7 8 9 10
b
Sucrose cushion Pellet samples
blot αFLAG
WT m2
– .5 1 2 – .5 1 2 mM MBS
250
150
100
75
50
37
25
20
M 1 2 3 4 5 6 7 8
c
blot αFLAG
FLAG Elute: WT Δ4 caf20Δ
1 mM MBS: – + + + + + +
250
150
100
75
50
37
25
20
15
M 1 2 3 4 5 6 7
d
FLAG Elute: WT Δ4 WT m2 ΔAC
1 mM BMH: – + + + +
250
150
100
75
50
37
25
20
15
αFLAG
< WT
< Δ4
< ΔAC
M 1 2 3 4 5

Figure S7

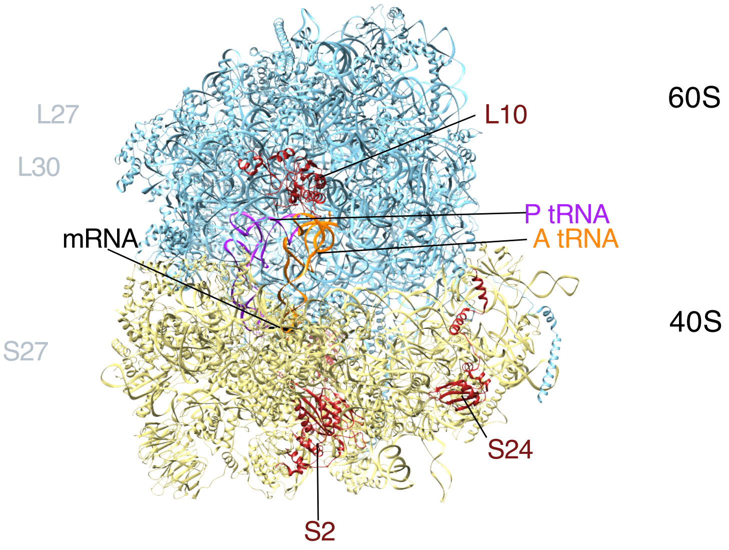

Figure S8

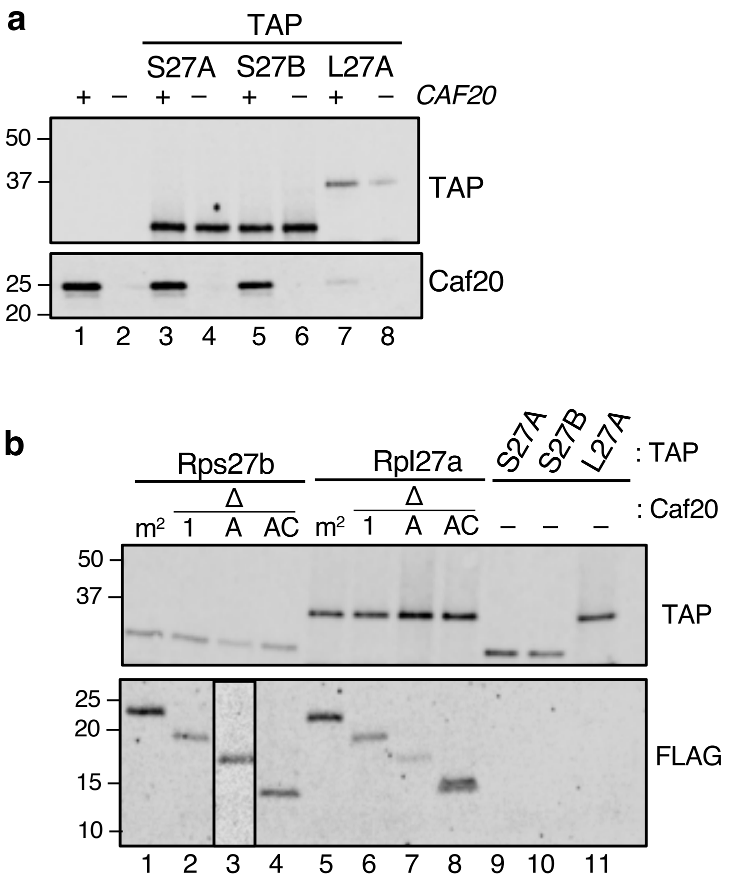
a
TAP
S27A
S27B
L27A
+ – + – + – + –
CAF20
50
37
TAP
25
20
Caf20
1 2 3 4 5 6 7 8
b
Rps27b
Rpl27a
S27A
S27B
L27A
: TAP
Δ
Δ
: Caf20
m2 1 A AC m2 1 A AC – – –
50
37
TAP
25
20
15
10
FLAG
1 2 3 4 5 6 7 8 9 10 11

Source data Figure 1c.

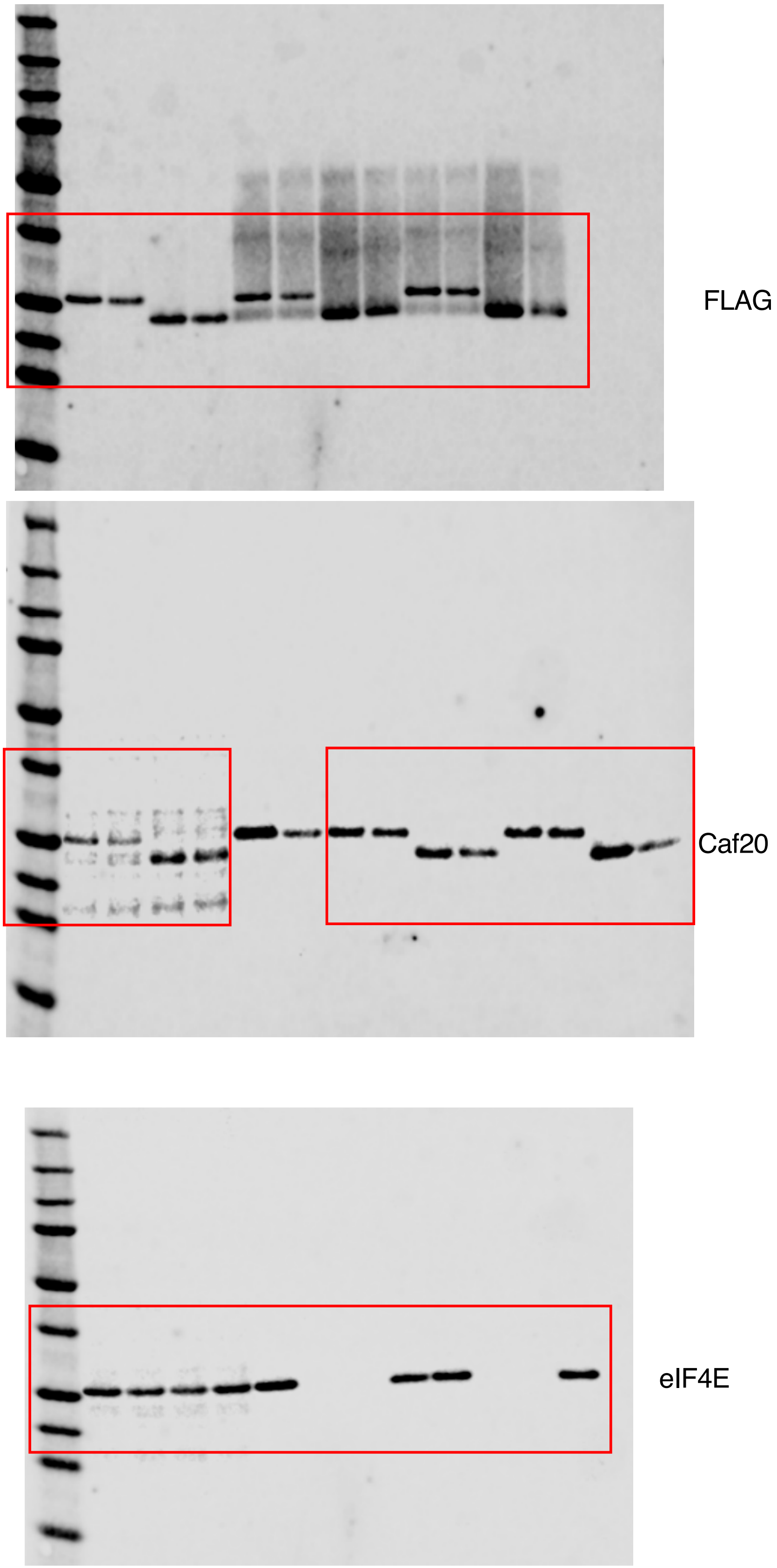

Source data Figure 2a

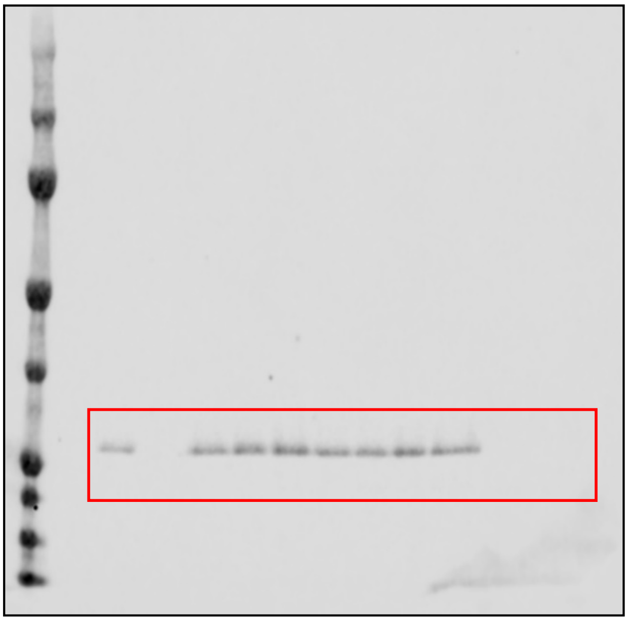

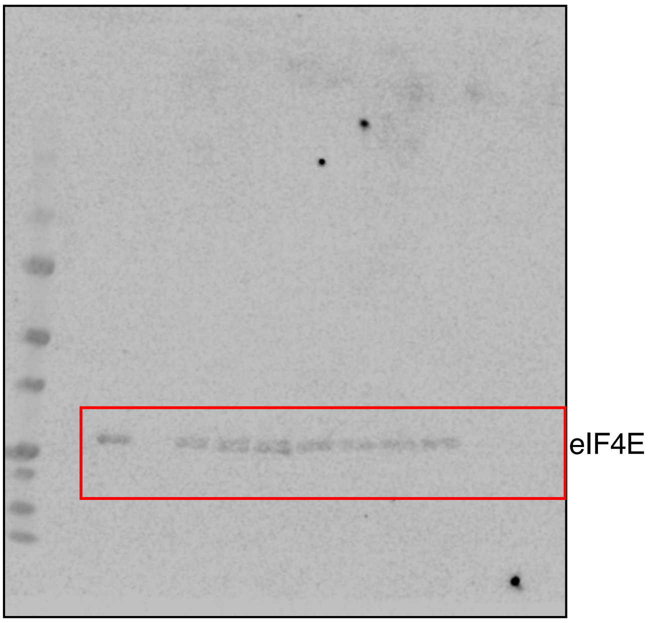

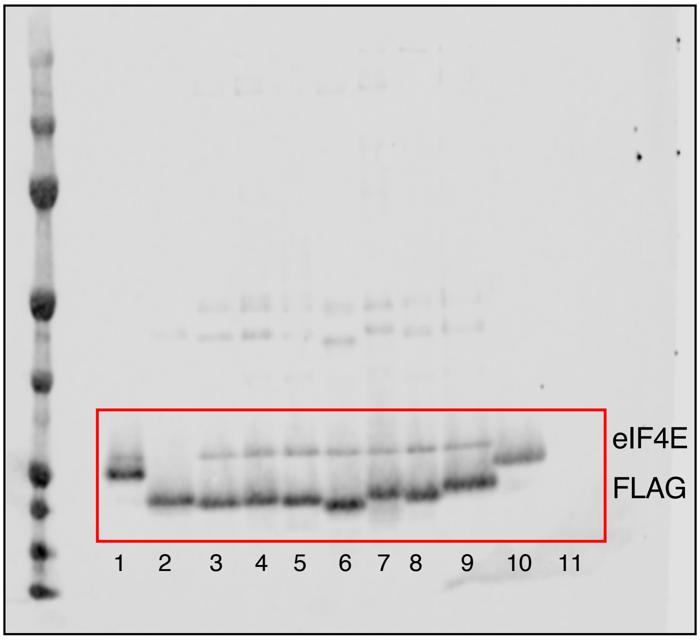

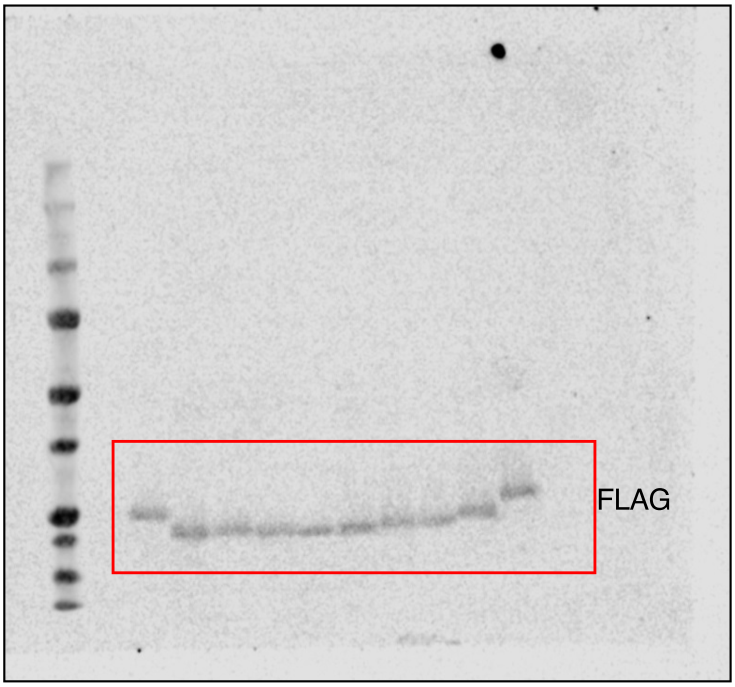

Source data Figure 2b

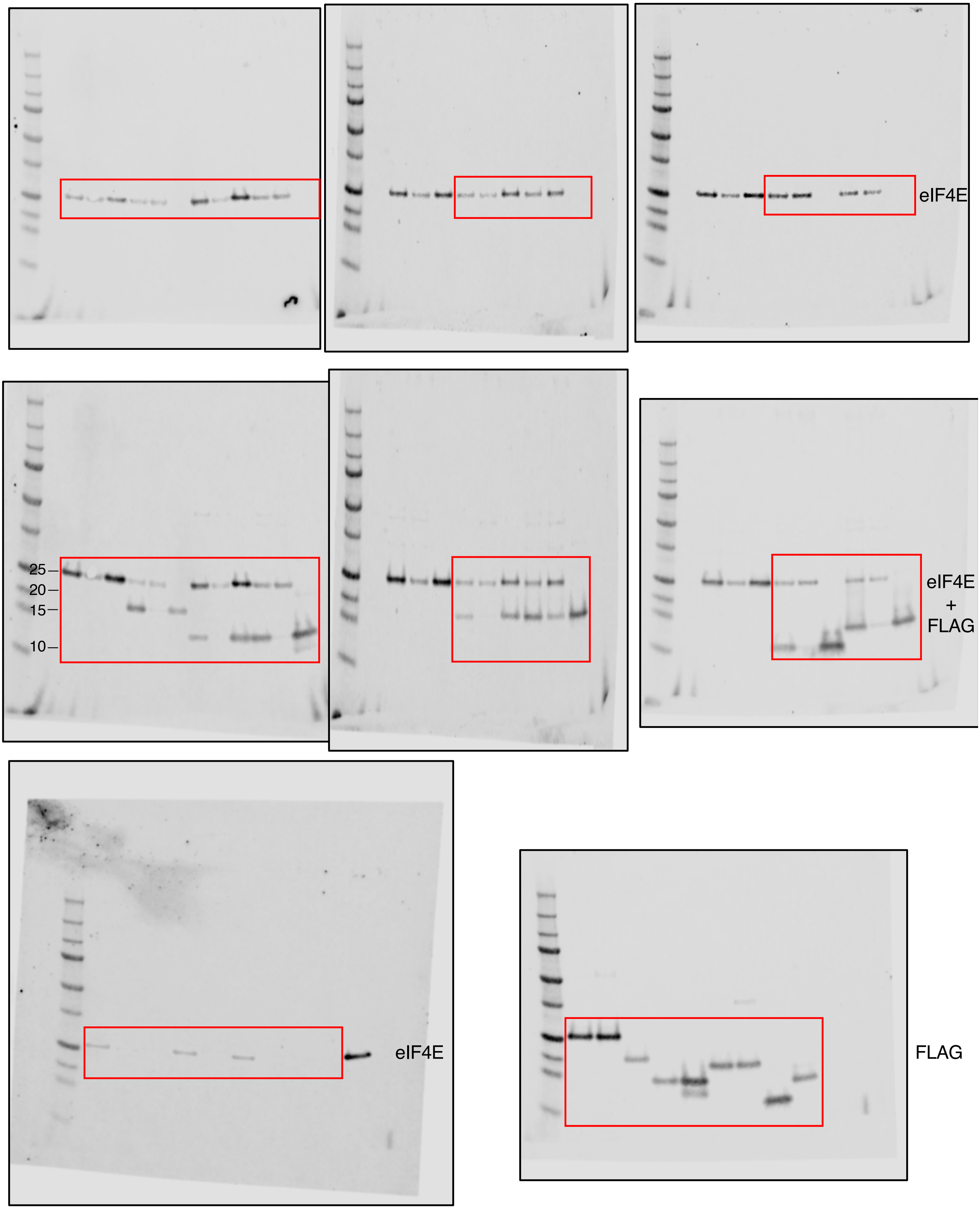

Source Data Figure 3b

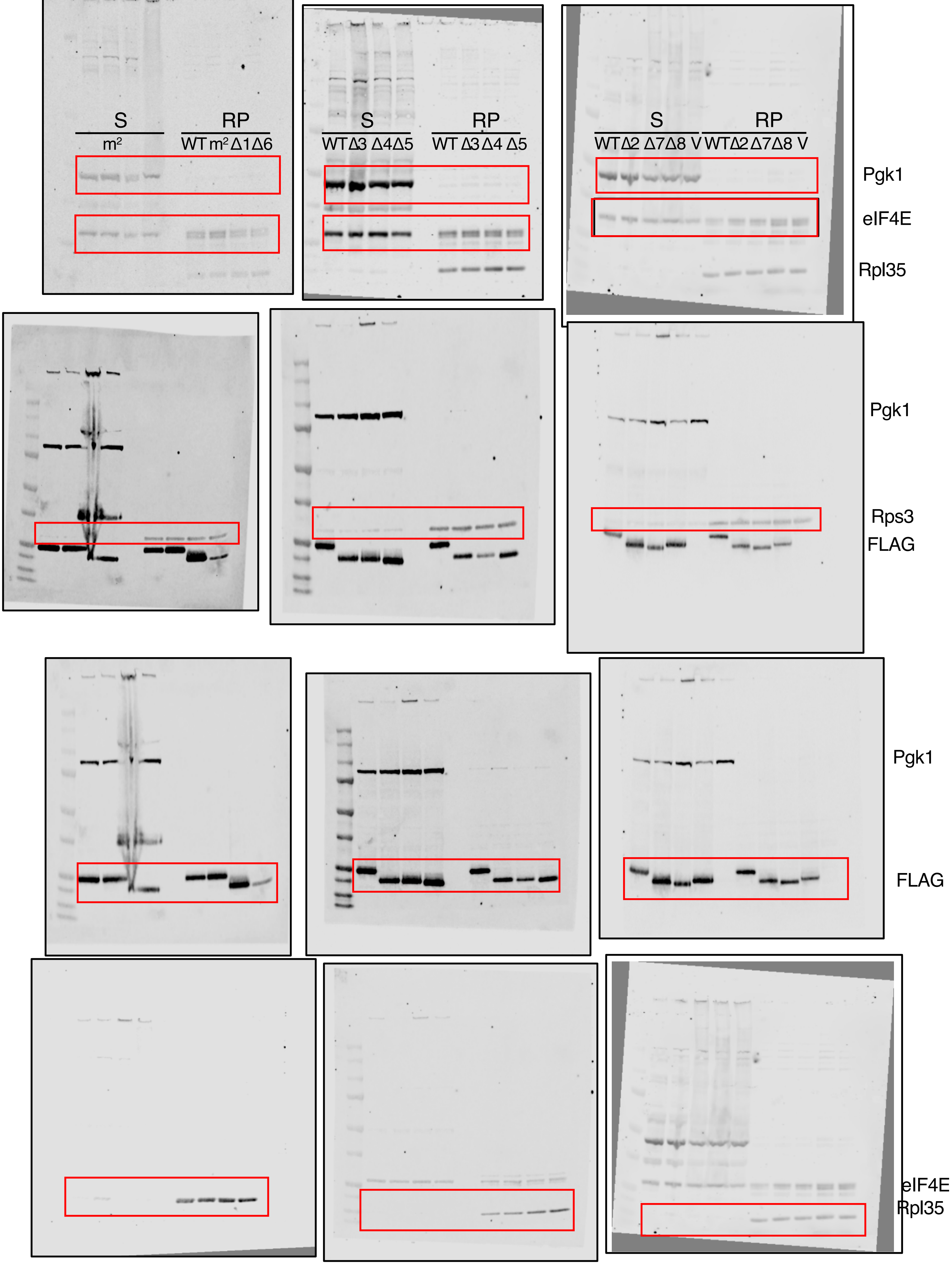

Source Data Figure 3c

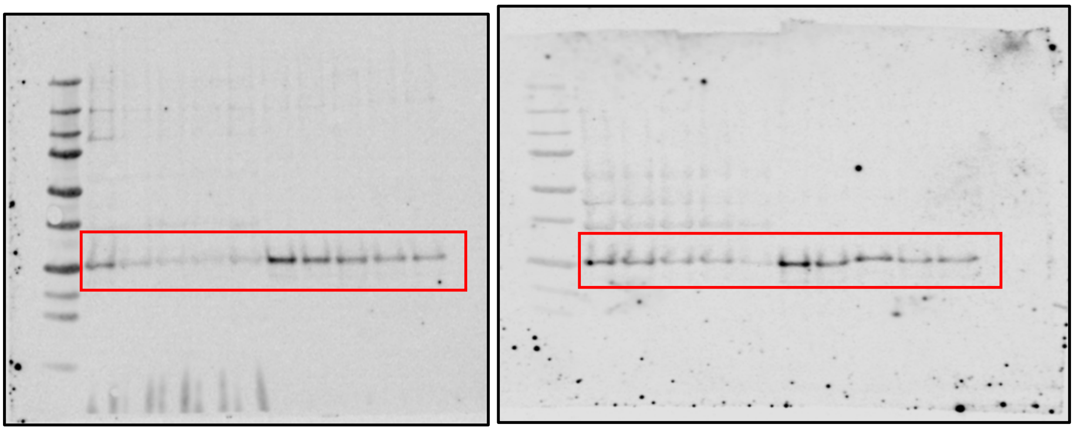

eIF4E

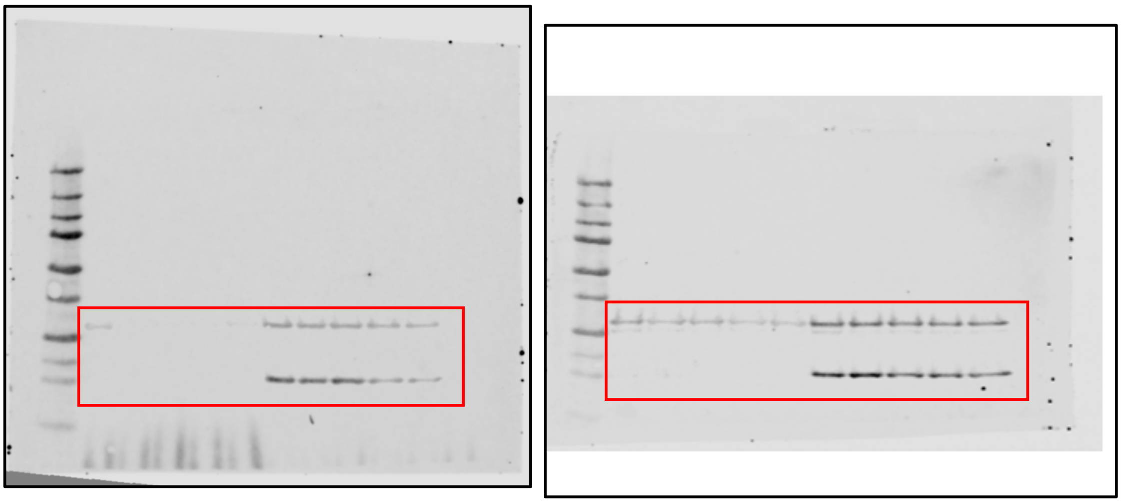

Rps3

Rpl35

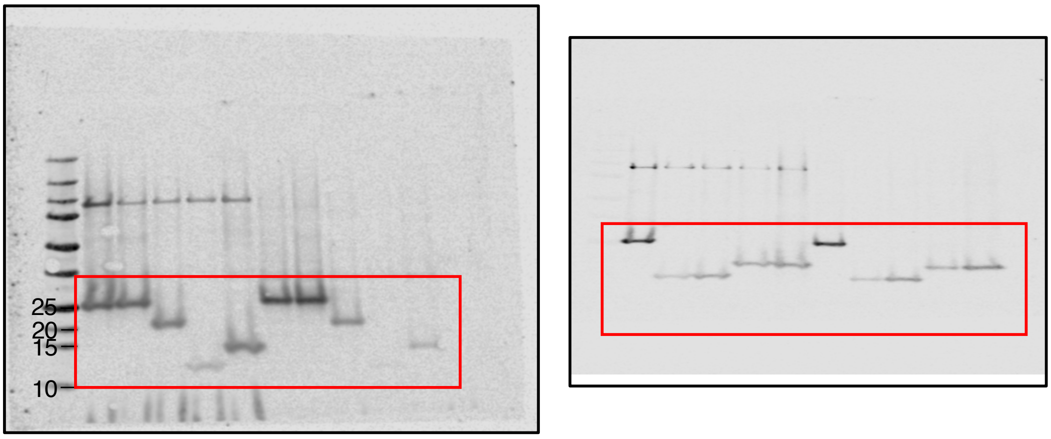

FLAG

Source data Figure 6

a

TAP-tag protein expression

αProtein A

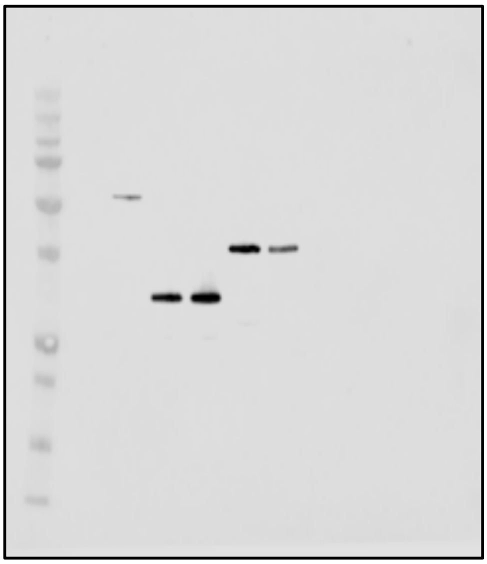

b Ribosome association and MBS reactivity

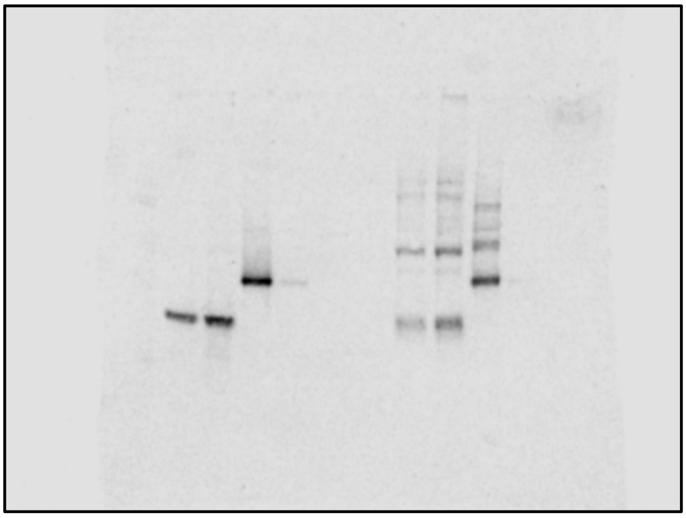

αProtein A

Source data Supplementary Figure S3

a

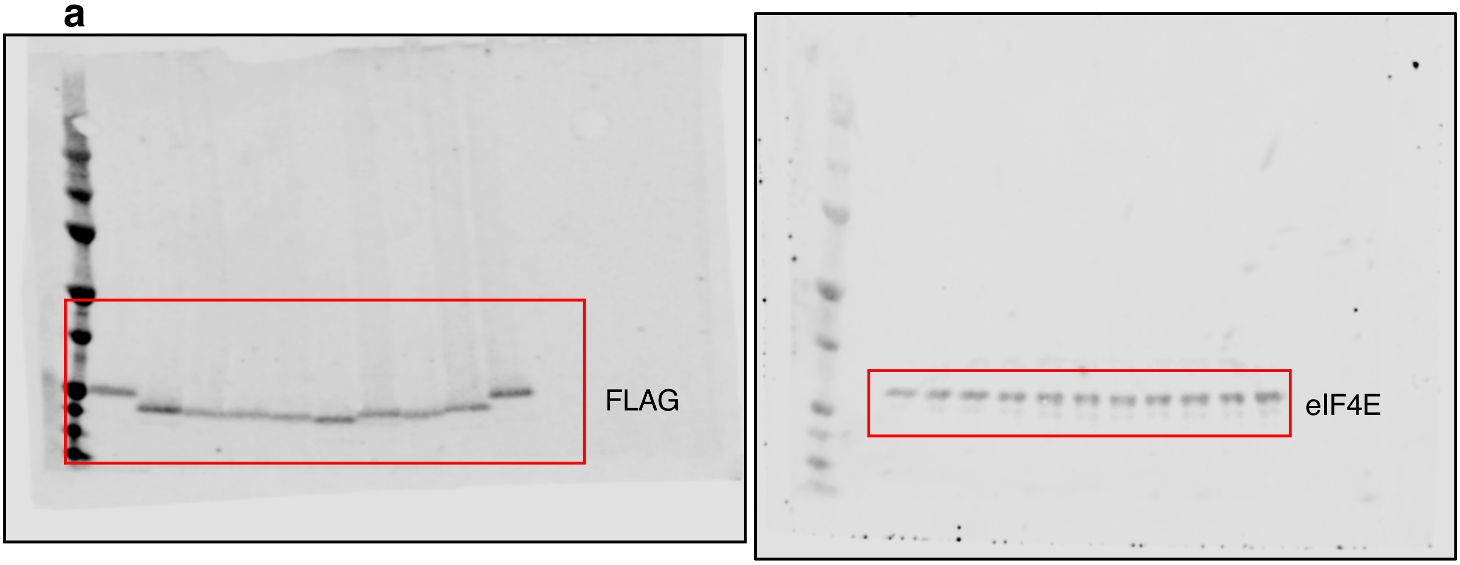

b

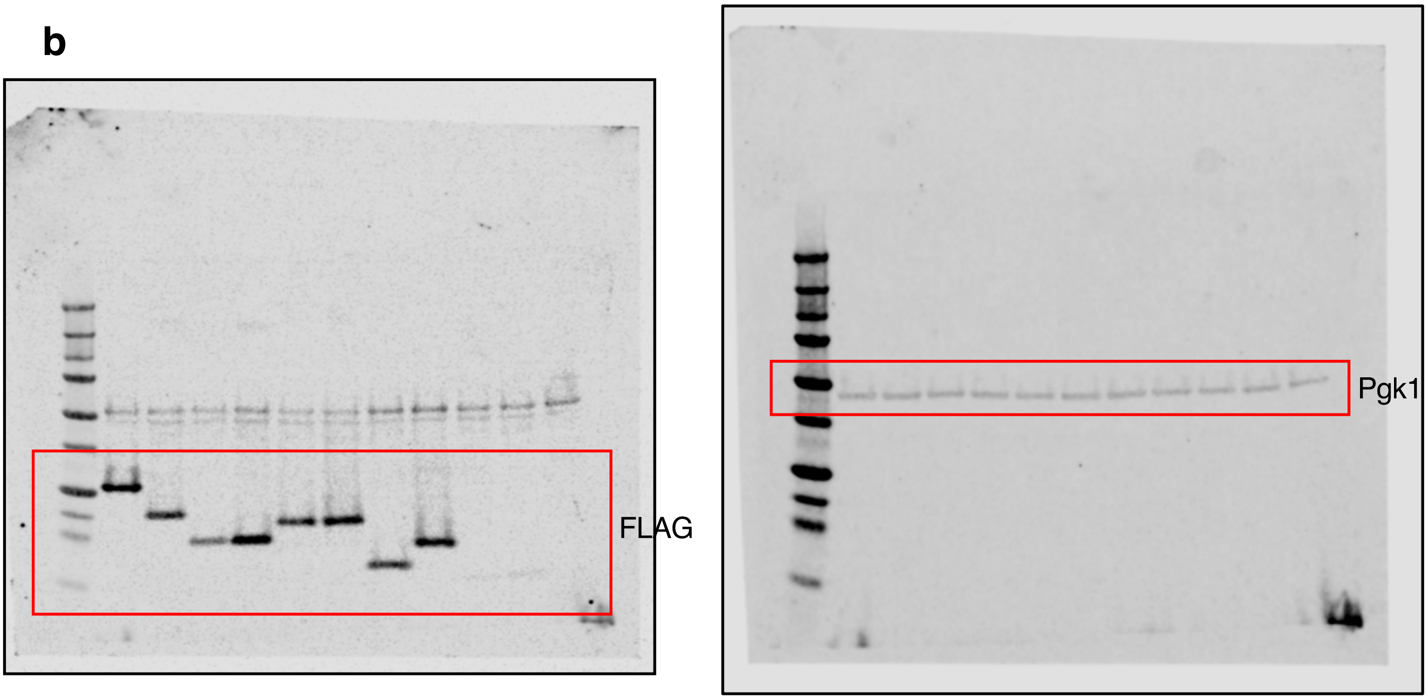

Source data Supplementary Figure S5

b

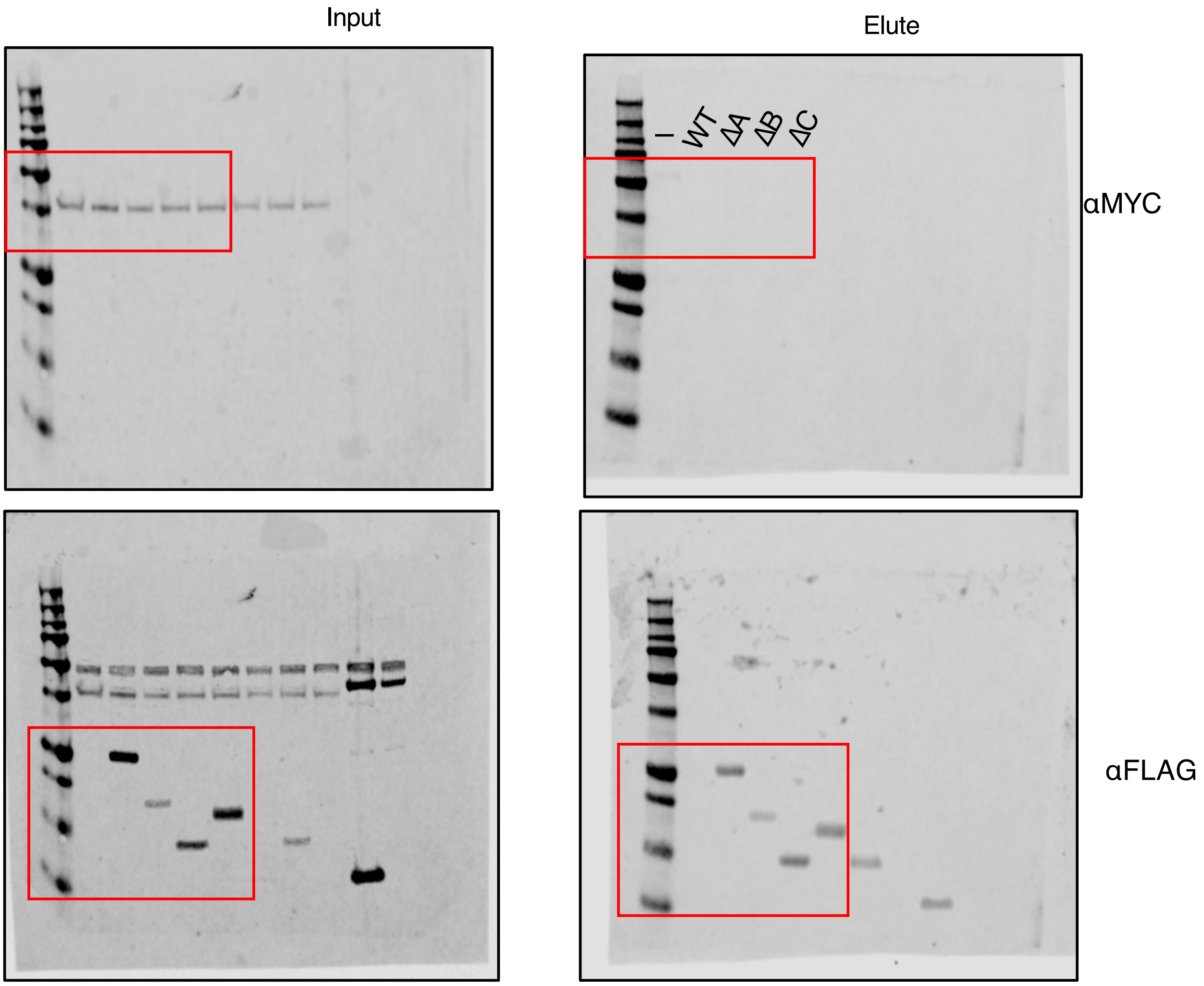

Source data Supplementary Figure S8

a

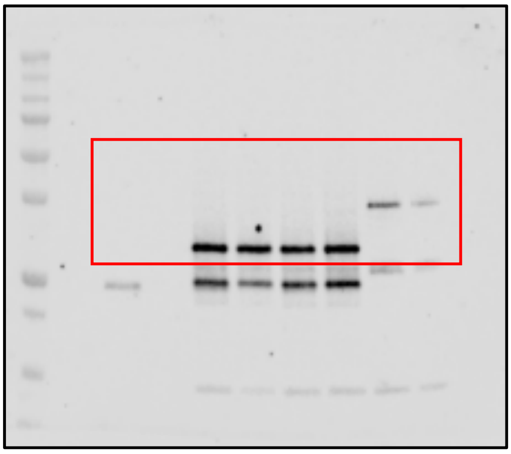

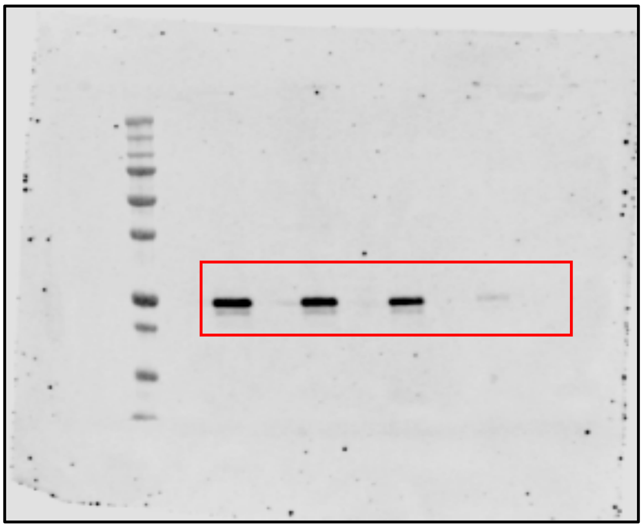

b

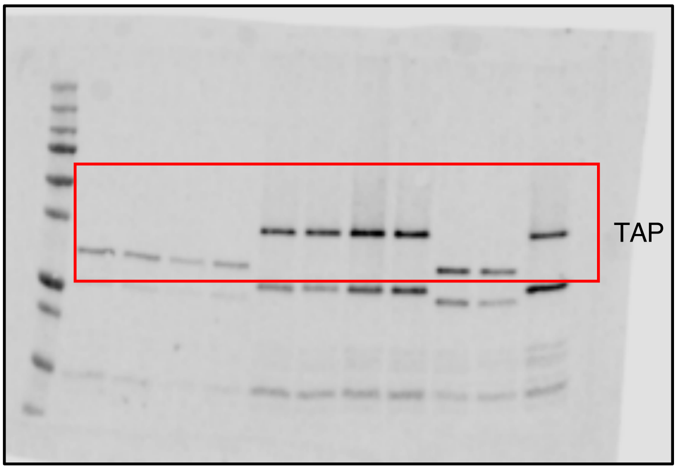

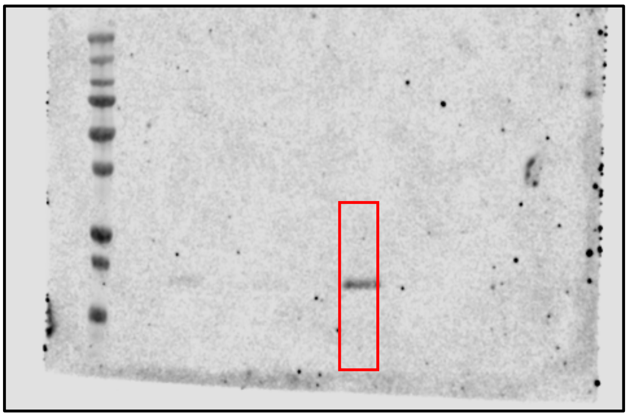

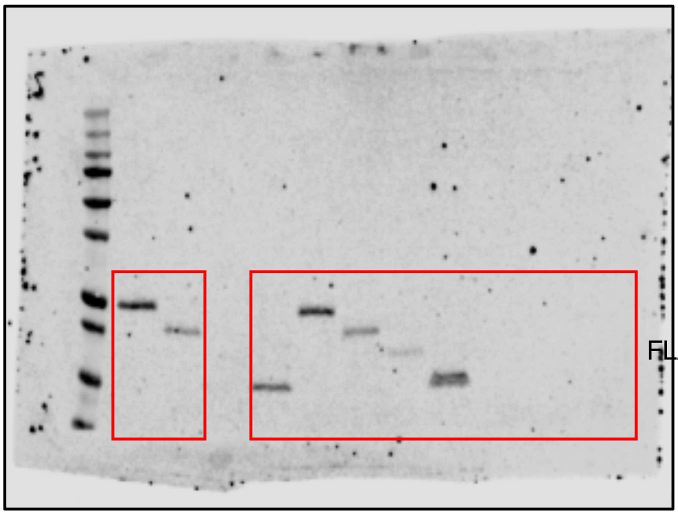

Supplement: Supplementary file 1 — Supplementary Information. [file 41598_2021_92931_MOESM1_ESM.pdf]
